# Preserving spatial and quantitative information in unpaired biomedical image-to-image translation

**DOI:** 10.1016/j.crmeth.2025.101074

**Published:** 2025-06-09

**Authors:** Joshua Yedam You, Minho Eom, Tae-Ik Choi, Eun-Seo Cho, Jieun Choi, Minyoung Lee, Changyeop Shin, Jieun Moon, Eunji Kim, Pilhan Kim, Cheol-Hee Kim, Young-Gyu Yoon

**Affiliations:** 1 School of Electrical Engineering, KAIST, Daejeon, Republic of Korea; 2 Department of Biology, Chungnam National University, Daejeon, Republic of Korea; 3 Graduate School of Medical Science and Engineering, KAIST, Daejeon, Republic of Korea; 4 KAIST Institute for Health Science and Technology, Daejeon, Republic of Korea; 5 IVIM Technology, Daejeon, Republic of Korea; 6 Department of Semiconductor System Engineering, KAIST, Daejeon, Republic of Korea

**Keywords:** image-to-image translation, multimodal imaging, information consistency, learnable upsampling, spatial alignment, quantitative information preservation, biomedical image synthesis, calcium imaging translation, virtual staining

## Abstract

Analysis of biological samples often requires integrating diverse imaging modalities to gain a comprehensive understanding. While supervised biomedical image translation methods have shown success in synthesizing images across different modalities, they require paired data, which are often impractical to obtain due to challenges in data alignment and sample preparation. Unpaired methods, while not requiring paired data, struggle to preserve the precise spatial and quantitative information essential for accurate analysis. To address these challenges, we introduce STABLE (spatial and quantitative information preserving biomedical image translation), an unpaired image-to-image translation method that emphasizes the preservation of spatial and quantitative information by enforcing information consistency and employing dynamic, learnable upsampling operators to achieve pixel-level accuracy. We validate STABLE across various biomedical imaging tasks, including translating calcium imaging data from zebrafish brains and virtual histological staining, demonstrating its superior ability to preserve spatial details, signal intensities, and accurate alignment compared to existing methods.

## Highlights

• STABLE is an unsupervised image-to-image translation algorithm for biomedical imaging
• STABLE preserves spatial and quantitative information from the original image
• STABLE can model complex, non-monotonic relationships across different image domains
• STABLE outperforms previous methods in a range of biomedical translation tasks

## Motivation

The ability to virtually translate between biomedical imaging modalities holds substantial potential for streamlining biomedical research and diagnostics by offering diverse representations of the same sample. However, supervised translation approaches require paired images, which are often impractical to obtain due to tissue degradation, sample alterations, or incompatible preparation methods. This limitation has led to the development of unpaired image-to-image translation methods. Yet, existing unpaired methods struggle to preserve the precise spatial and quantitative information crucial for biomedical applications, often resulting in misaligned or inaccurate translations. To address these challenges, we introduce STABLE (spatial and quantitative information preserving biomedical image translation), an unpaired image-to-image translation method that enables accurate preservation of spatial alignment and quantitative signals, facilitating reliable multimodal integration of biomedical imaging data.

## Graphical abstract

**Figure:**
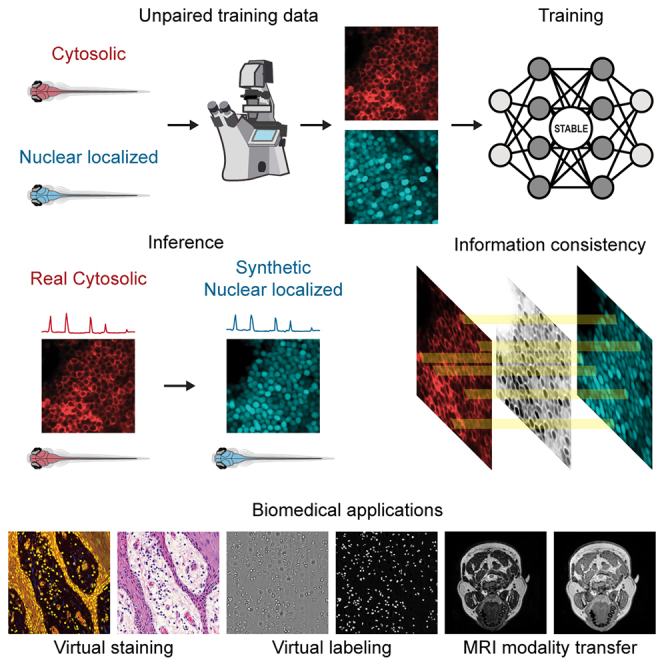

## Teaser

You et al. propose an unpaired image-to-image translation algorithm for biomedical imaging. They demonstrate that the integration of feature-level consistency with learnable feature upsampling enables the accurate preservation of spatial and quantitative information in a range of biomedical tasks and modalities, including complex, non-monotonic transdomain relationships.

## Introduction

Biomedical imaging includes various modalities, each designed to visualize different aspects of biological processes. The use of various staining and imaging techniques, such as immunofluorescence^1^^,^^2^ and histological staining,^3^^,^^4^ is essential in research and clinical practice, providing valuable insights into structures and functions at the cellular and tissue levels. Naturally, the ability to virtually translate between these imaging modalities holds substantial potential for streamlining biomedical research and diagnostics by offering diverse representations of the same biological sample.^5^^,^^6^^,^^7^^,^^8^^,^^9^^,^^10^^,^^11^^,^^12^

Early image translation methods have employed supervised learning approaches, which require paired images from both modalities.^13^ These approaches have been applied to translate between various imaging modalities, demonstrating success in tasks such as converting transmitted-light microscopy images into antibody-stained fluorescence,^5^^,^^11^ generating super-resolution images from their lower-resolution counterparts,^14^^,^^15^^,^^16^ and creating virtual histological stains from autofluorescence images.^17^

Despite their potential, supervised learning approaches face inherent limitations that hinder their widespread adoption by biologists and clinicians. The primary challenge lies in acquiring paired images from different modalities, which is often impractical due to tissue degradation, sample alterations during imaging, or incompatible preparation methods. Additionally, variations in positioning, light power, or staining quality between sessions introduce discrepancies, complicating efforts to obtain paired images.^8^^,^^18^^,^^19^^,^^20^

To address these limitations, researchers have turned to unpaired image-to-image translation methods,^21^^,^^22^^,^^23^^,^^24^ such as CycleGAN.^25^ CycleGAN can translate images from one modality to another, generating results that closely resemble the target modality while loosely retaining the spatial structure of the original image. This preservation of spatial structure is enforced by the cycle consistency constraint, which requires that a translated image can be mapped back to the original input. This would not be possible if structural information were lost during translation.

However, while effective in preserving spatial structure for general computer vision applications, CycleGAN and similar methods often fail to maintain the precise spatial and quantitative information required for biomedical applications, which require pixel-level accuracy to properly align signal and structural elements across multiple modalities. This is because the cycle consistency constraint only ensures that the translated image retains enough information for reconstruction, which does not necessarily lead to an accurate translation. This limitation can result in issues such as inverted foreground and background mappings or spatial misalignment in the translated image, which are unacceptable for applications where the preservation of exact spatial and quantitative information is crucial.^24^

Subsequent unpaired translation approaches have been proposed. MUNIT^26^ decomposes images into a shared content code and a variable style code, enabling diverse multimodal outputs by sampling different styles. However, its content encoder compresses spatial information to a low resolution, and stochastic style injections can disrupt fine-grained structure and introduce variability that undermines reproducibility and quantitative consistency. SANTA^27^ introduces a shortest-path regularization to address translation ambiguity by constraining translation mappings to follow the shortest paths along a latent space. However, it lacks explicit constraints that ensure fine spatial alignment, making it insufficient for applications that require such a level of precision.

Efforts to address these limitations have led to the development of biomedical-specific methods such as UTOM,^24^ which extend the CycleGAN framework for optical microscopy. UTOM introduces a saliency consistency constraint to handle monotonic relationships in pixel intensities between input and output images. However, it struggles in cases where the monotonicity assumption is violated, as shown in the following sections. Moreover, UTOM falls short in tasks requiring pixel-level accurate spatial alignment, emphasizing the need for more robust methods capable of preserving spatial and quantitative information.

To overcome these challenges, we introduce STABLE (spatial and quantitative information preserving biomedical image translation), an unpaired image-to-image translation algorithm specifically designed to preserve the spatial and quantitative details essential for biomedical applications. STABLE addresses this key requirement by enforcing information consistency of the full resolution features and incorporating learnable dynamic upsampling operators. We demonstrate STABLE’s performance across a range of tasks, including calcium imaging translation of zebrafish neurons, virtual histological staining, virtual fluorescent labeling, magnetic resonance imaging (MRI) modality transfer, denoising, and super-resolution. Our results show that STABLE consistently outperforms existing methods in preserving both spatial and quantitative information, such as cell location, signal intensity, and fine structural details.

## Results

### STABLE: Spatial and quantitative information preserving unpaired image-to-image translation

STABLE aims to preserve spatial and quantitative information in unpaired biomedical image translation by addressing the inherent limitations of image reconstruction imposed by the cycle consistency constraint. Existing methods that utilize the cycle consistency term^25^ heavily rely on image reconstruction for information preservation. While this term encourages the translated image to resemble the input image, it fundamentally only requires that sufficient information is preserved for reconstruction rather than ensuring the accurate preservation of spatial and quantitative details. Consequently, errors in translation, such as spatial misalignments or incorrect intensity mappings, may not conflict with this constraint as long as they can be reverted in the reconstruction stage.

**Figure 1:**
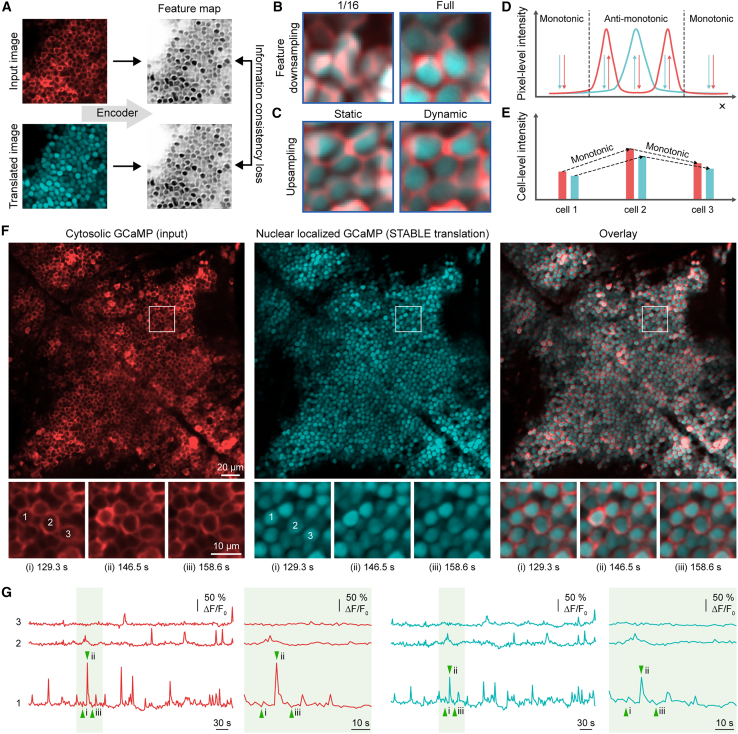
Overview of the proposed method (A) Schematic representation of the proposed information consistency constraint. Encoders from each image domain extract common feature information and generate feature maps that maintain the same spatial resolution as the input image. The information consistency loss is minimized to enforce similarity between the feature maps. (B) Impact of feature map size on translation accuracy. From left to right: translation overlay with feature map of full spatial resolution and translation overlay with feature map of 1/16 spatial resolution. When the feature map extracted by the encoder retains the same spatial resolution as the input image (full), the translated image achieves high spatial fidelity compared to when the feature map is downsampled to 1/16 of the input image size. (C) Comparison of results with and without dynamic upsampling. Shown are the overlays of the input and translated images for the calcium imaging task. The input cytosolic GCaMP is labeled red, and the translated nuclear-localized GCaMP in cyan. From left to right: translation overlay without dynamic upsampling (bilinear interpolation) and with dynamic upsampling. The right image demonstrates the enhanced spatial alignment with dynamic upsampling, compared to the left image that used fixed, static bilinear upsampling. (D) Plot illustrating the change in pixel-level intensity along the spatial axis (*x* axis) for the cytosolic (blue curve) and corresponding nuclear-localized signals (red curve). Within the neuronal cell body (region between the dashed vertical lines), the two domains show anti-monotonic relationships: as the cytosolic signal increases in intensity along the axis, the nuclear-localized signal shows an inverse relationship, decreasing in intensity. Outside of the cell (i.e., in the background), the intensity relationship is monotonic, where both cytosolic and nuclear-localized signals decrease in intensity. (E) Plot illustrating the difference in cell-level intensity. At the cell-level, the intensity shows a monotonic, linear relationship between the cytosolic and nuclear-localized signals. As the overall calcium signal intensity increases within the cell (blue bars for cytosolic and red bars for nuclear-localized signals), both cytosolic and nuclear-localized signals increase, demonstrating that the intensity changes in both compartments are positively correlated at the cell level. (F) Calcium imaging data translation results of STABLE. From left to right: input cytosolic GCaMP (red), translated nuclear-localized GCaMP (cyan), and overlay of the input and translated images. Magnified views of the boxed regions are shown along the bottom, illustrating temporal changes at specific time points (129.3, 146.5, and 158.6 s). Scale bar for large views, 20 μm. Scale bar for magnified views, 10 μm. (G) Each row presents the corresponding neuronal activity traces for three selected cells from (F), with cytosolic input traces (red) on the left and nuclear-localized translated traces (cyan) on the right, aligned in time.

These limitations necessitated the development of information consistency constraints in STABLE. In addition to employing cycle consistency, STABLE enforces the consistency of information in a separate feature domain that acts as a bridge between the two domains. This feature domain captures spatial and quantitative information common to both domains. To achieve this, STABLE employs feature encoders that map images into the shared feature domain (Figure 1A). The information consistency term is applied to minimize the difference between the feature maps of the input and the translated images, allowing for information common to both image domains to be preserved across translation. By combining this information consistency with the cycle consistency, STABLE captures complex spatial structures, such as cell locations and intensity variations.

To achieve pixel-level alignment between translated and input images, it is critical for the feature domain to retain precise spatial information from the input images. To implement this, STABLE’s generators are decomposed into encoder-decoder pairs designed to learn to encode consistent feature maps across the translation pipeline (Figure S1A). The encoders $G_{A}$ and $G_{B}$ extract corresponding feature maps $Z_{A}\in R^{C_{Z}\times H\times W}$ and $Z_{B}\in R^{C_{Z}\times H\times W}$ from the input images $X_{A}$ and $X_{B}$ from image domains A and B, respectively, where *H* and *W* represent the height and width of the input image:

$$Z_{A}=G_{A}\left(X_{A}\right),Z_{B}=G_{B}\left(X_{B}\right)\text{.}$$

Importantly, these feature maps are kept at full resolution to preserve the pixel-level spatial information from the input images, enabling precise alignment of biological features such as cell shapes and positions (Figure 1B). The feature maps are then translated to the target domains via domain-specific decoders $F_{A}$ and $F_{B}$:

$$X_{A\to B}=F_{B}\left(Z_{A}\right),X_{B\to A}=F_{A}\left(Z_{B}\right)\text{.}$$

The information consistency loss $L_{info}$ is defined to minimize the differences between the feature maps of the input and translated images and enforces the maintenance of spatial and quantitative information across the two domains (Figure S1A):

$$L_{info}\left(G_{A},G_{B},F_{A},F_{B}\right)={\left|Z_{A}-G_{B}\left(X_{A\to B}\right)\right|}_{1}+{\left|Z_{B}-G_{A}\left(X_{B\to A}\right)\right|}_{1}\text{.}$$

To encode feature maps with the same spatial resolution as the images, we designed the encoder and decoders in a multi-scale manner, where we downsampled and then upsampled intermediate feature maps back to the original resolution (Figure S1C). Many existing multi-scale architectures use fixed operations such as bilinear interpolation to upsample the intermediate feature maps, which can result in spatial misalignments due to their non-learnable nature. The repetitive application of such upsampling methods can accumulate spatial errors throughout the network, leading to misalignment in the translated images.^28^^,^^29^^,^^30^ To mitigate these issues, STABLE incorporates learnable dynamic upsampling operators^31^ to replace conventional upsampling operations (Figure S1D). This approach allows the model to adaptively learn to upsample feature maps to accurately preserve spatial information such as the boundaries, shapes, and locations of biological structures during upsampling, as shown in Figure 1C, where the translated features (cyan) correctly align with the input structural elements (red), unlike the misalignments observed with fixed upsampling implementations.

The dynamic upsampling operator adaptively learns offsets for the sampling grid, allowing the feature maps to be resampled accurately. Given a low-resolution feature map $X\in R^{C\times H\times W}$, the upsampling operator predicts the offsets using a 2D convolutional layer with a 1 × 1 kernel applied to the input feature map X, which is followed by pixel shuffling^32^ to produce an offset grid $O\in R^{2\times sH\times sW}$, where s is the upsampling factor. These offsets adjust the sampling grid $G\in R^{2\times sH\times sW}$ to yield the final sampling positions $S\in R^{2\times sH\times sW}$: S=G+O. These sampling positions are used to resample the original feature map, generating the upsampled feature map $X^{\prime }\in R^{C\times sH\times sW}$ (see the STAR Methods).

### Performance evaluation of STABLE on calcium imaging data

To validate the effectiveness of STABLE in preserving spatial and quantitative information across translation, we selected a dataset of *in vivo* calcium imaging data from zebrafish brains. This dataset consists of confocal microscopy recordings of neurons in the zebrafish brain expressing GCaMP either in the cytosol or in the nucleus.^33^^,^^34^^,^^35^^,^^36^^,^^37^^,^^38^ In particular, we chose the translation task of converting images of neurons expressing GCaMP in the cytosol to images of neurons expressing GCaMP in the nucleus. This task was chosen for its inherent complexity, which requires the translation network to simultaneously model intensity relationships at two distinct levels of abstraction: the pixel level and cell level. Specifically, at the pixel level, intensity relationships between the cytosolic GCaMP and nuclear-localized GCaMP vary depending on spatial location (Figure 1D). Within the cell, the relationship is anti-monotonic, where the strong cytosolic signals at the edges of the cell body correspond to weak nuclear-localized signals, and vice versa at the center. Meanwhile, outside the cell (i.e., in the background), the relationship is monotonic, with positively correlated intensities across domains. At the cell level, the intensity relationship becomes monotonic, which means that the signals of the neurons must be positively correlated (Figure 1E).

The coexistence of different intensity relationships at distinct levels of abstraction makes this translation task particularly difficult for handcrafted constraints, such as the saliency consistency term used in UTOM.^24^ This term enforces that the masks thresholded by the intensity from the input and translated images are consistent, which assumes a monotonic intensity relationship between image domains. However, no single binary map can simultaneously represent the spatial structures in both pixel-level and cell-level domains in this task. Conceptually, to achieve accurate translation, the model must identify each neuron in the cytosolic GCaMP image, measure the brightness along the edge of each neuron, and translate that brightness to appear at the nucleus in the translated image. Given these complexities, this dataset offers a rigorous benchmark for assessing the image translation performance, where the ability to accurately perform such a challenging task demonstrates STABLE’s potential to extend to other biomedical imaging tasks, especially those where preserving fine biological details is critical.

We generated the training dataset by extracting patches from each image domain, forming separate datasets for cytosolic and nuclear-localized neurons. From the calcium imaging recordings, we randomly sampled frames and cropped them to create image patches of 1 (t) × 256 (*x*) × 256 (*y*) pixels used for training the model. Since the cytosolic and nuclear-localized samples are from different animals, the dataset is unpaired, with no direct correspondence between source and target images. We train on a subset of the dataset, with all method evaluations conducted on samples that the model had not seen during training. Additional methodological details regarding the dataset preparation and training are provided in the STAR Methods section.

We compared STABLE against baseline unpaired image-to-image translation methods: CycleGAN,^25^ MUNIT,^26^ SANTA,^27^ and UTOM.^24^ CycleGAN, MUNIT, and SANTA are methods developed for general computer vision tasks, whereas UTOM is designed for translating optical microscopy images, which adds a saliency consistency term to CycleGAN. Our results demonstrated that all of the baseline methods failed to conserve the location and signal intensities of neurons, whereas STABLE successfully maintained these features.

To conduct a qualitative comparison, we evaluated how STABLE and the baseline methods translated cytosolic neurons to nuclear-localized neurons by overlaying the input cytosolic images with their corresponding translated nuclear-localized images. The goal of this comparison was to determine whether the translated nuclear-localized neurons were correctly positioned within the cytosolic regions without overlap between the cytosol and the nucleus. Ideally, the nuclear-localized neurons should be centrally located within the cytosol, with no misalignment or overlap at the boundaries (Figures 1F and 1G; Video S1).

**Figure 2:**
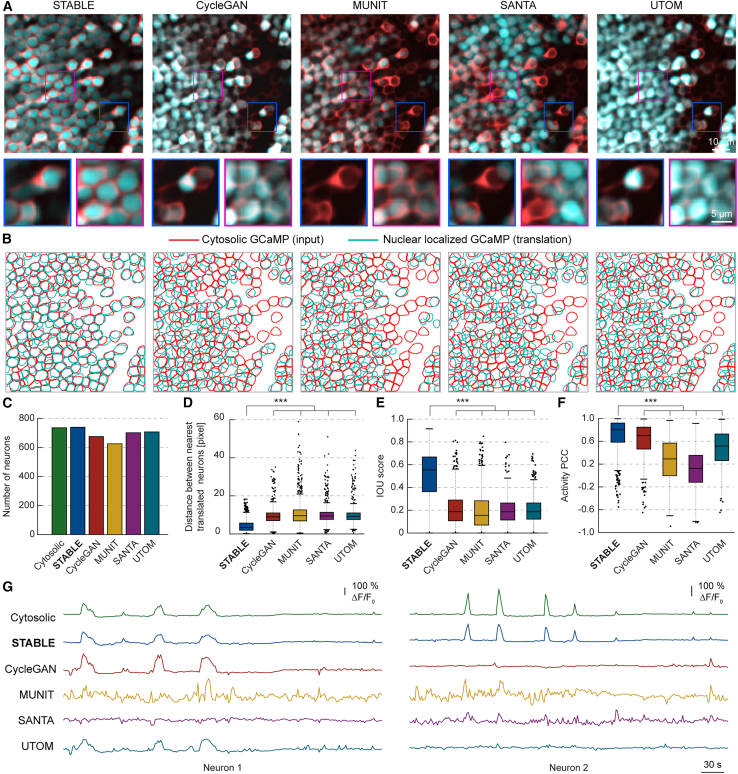
Quantitative comparison of STABLE with other unpaired image-to-image translation methods in calcium imaging (A) STABLE compared with unpaired image-to-image translation methods in the calcium imaging translation task, converting cytosolic GCaMP to nuclear-localized GCaMP. From left to right: STABLE, CycleGAN, MUNIT, SANTA, and UTOM. Input cytosolic GCaMP (cyan) is overlaid with the translated nuclear-localized GCaMP (red). Magnified views of the boxed regions are presented along the bottom. Scale bar for large views, 10 μm. Scale bar for magnified views, 5 μm. (B) Segmented regions of interest (ROIs) of the input cytosolic GCaMP (cyan) overlaid with the translated nuclear-localized GCaMP (red). ROIs were obtained using Cellpose segmentation models applied to input and translated images. (C) Comparison of the number of neuron ROIs between the input cytosolic GCaMP (green) and the translated nuclear-localized GCaMP. (D) Box-and-whisker plot showing the distance between the nearest input and translated neurons. Distances were measured using the L2 distance between the center of mass of each neuron instance segmentation. A two-sample t test was used (*n* = 736 for each test, representing the number of input cytosolic neurons; n.s. *p* > 0.05, ∗*p* ≤ 0.05, ∗∗*p* < 0.01, and ∗∗∗*p* < 0.001). (E) Box-and-whisker plot comparing the intersection over union (IoU) scores between input and translated ROIs. A two-sample t test was performed (*n* = 736 for each test, representing the number of input cytosolic neurons; n.s. *p* > 0.05, ∗*p* ≤ 0.05, ∗∗*p* < 0.01, and ∗∗∗*p* < 0.001). (F) Box-and-whisker plot comparing Pearson correlation coefficients (PCCs) of neuronal activity traces between the nearest input and translated neurons. A two-sample t test was performed (*n* = 736 for each test, representing the number of input cytosolic neurons; n.s. *p* > 0.05, ∗*p* ≤ 0.05, ∗∗*p* < 0.01, and ∗∗∗*p* < 0.001). (G) Neuronal activity traces from two selected neurons are visualized and compared across methods and the cytosolic input (green). From top to bottom: input cytosolic, STABLE, CycleGAN, MUNIT, SANTA, and UTOM.

Video S1. Calcium imaging data translation, related to Figures 1 and 2

STABLE successfully achieved this by accurately positioning the nuclear-localized neurons in the center of the cytosolic regions, ensuring there was no overlap between the two compartments. In contrast, the baseline methods—CycleGAN, MUNIT, SANTA, and UTOM—failed to achieve this level of precision (Figure 2A). Their translations often resulted in large overlaps, with nuclear-localized neurons mispositioned toward the edges or intersections of the cytosolic regions. This misalignment created unnatural overlaps between the nucleus and cytosol, indicating an inaccurate translation. Furthermore, STABLE maintained the signal intensity of the neurons during the translation process, preserving both the spatial and quantitative information from the input cytosolic image, whereas the baseline methods exhibited inconsistencies in this regard.

We also visualized the learned feature maps of STABLE, where the feature maps reveal that STABLE preserved the spatial information through clearly defined cellular structures and edges, while the quantitative signal information is maintained in various representations within the cellular boundaries (Figure S1B). These visualizations demonstrate how STABLE effectively captures common neuronal features from both domains, illustrating how the feature maps facilitate complex spatial localization and intensity conservation during the translation process.

To further evaluate STABLE’s performance, we analyzed segmented labeled regions of interest (ROIs) from the input and translated images to assess the accuracy of the translated neurons (Figure 2B). This involved using the Cellpose 3^39^ segmentation model to segment individual neurons from both image domains. The overlays of the ROI outlines in Figure 2B confirmed that STABLE preserved spatial information more effectively than the baseline methods, with its translated neurons showing greater consistency in both location and shape. We quantified the results by comparing the number of detected neuron ROIs between the input and translated images (Figure 2C). The results showed that STABLE most accurately preserved the number of detected neurons (*n* = 739) compared to the baseline methods (CycleGAN: 675, MUNIT: 625, SANTA: 701, and UTOM: 707), closely matching the count (*n* = 736) in the original cytosolic images. The baseline methods either overestimated or underestimated the number of neurons due to incorrectly placing the nuclei at the highly intense cell boundaries of the densely packed input cytosolic neurons. Next, we compared the distances between the input and translated neurons (Figure 2D). Distances were measured by computing the L2 distance between the center of mass of each neuron instance segmentation, where the average diameter of a single neuron is around 16 pixels. The results showed that STABLE excelled in maintaining the precise location of neurons, as evidenced by the lowest median distance (3.30) compared to the baseline methods: CycleGAN (8.98), MUNIT (9.58), SANTA (9.41), and UTOM (9.20). We also compared the intersection over union (IoU) score, which measures the overlap between the segmented regions in the input and translated images. STABLE scored the highest median IoU score of 0.554 compared to other methods: CycleGAN (0.188), MUNIT (0.155), SANTA (0.186), and UTOM (0.187) (Figure 2E).

Next, we validated the accuracy of the neuronal signals of the translated neurons by comparing the Pearson correlation coefficients (PCCs) of translated neuronal activity traces from the nearest input cytosolic neurons. STABLE most accurately preserved the signals, with a median PCC of 0.803, compared to CycleGAN (0.699), MUNIT (0.291), SANTA (0.124), and UTOM (0.518) (Figure 2F). We also qualitatively inspected the temporal neuronal activity traces from two neurons visualized and compared across methods (Figure 2G). The translated traces by STABLE were the most consistent compared to the baseline methods, which failed to preserve the features of the calcium signals in terms of shape, scale, and peaks. These results indicate that STABLE could accurately disentangle the signal of the cell from the structural information and preserve the neuronal activity across translations, which is crucial for potential downstream tasks such as spike detection.

To evaluate the performance contributions of each key component of STABLE, we conducted an ablation study by systematically adding each component from the baseline configuration (configuration 1) of only adversarial loss and cycle consistency loss terms. Configuration 2 builds on configuration 1 by adding the dynamic upsampling operator. Configuration 3 added the information consistency loss term to configuration 1. Configuration 4 represented the full implementation of STABLE, incorporating adversarial loss, cycle consistency loss, information consistency loss, and dynamic upsampling.

Qualitative inspection of the translation overlays (Figure S2A) indicated that the translations from configurations 1 and 2 resembled those of the baseline methods, inaccurately placing nuclear-localized neurons at the edges and intersections of the input cytosolic neurons. The addition of the information consistency loss term in configuration 3 slightly improved the accuracy of the spatial localization of neurons, although there was still some offset in neuron positions, most likely attributable to the use of a fixed upsampling operator. Configuration 4, which introduced the dynamic upsampling operator to Configuration 3, showed a marked improvement in the spatial alignment of neuron locations.

Consistent with the qualitative inspection, our quantitative analysis confirmed the progressive improvement across configurations. The addition of the information consistency loss term in configuration 3 improved spatial accuracy, reducing the median distance between the nearest translated neurons (7.01) compared to configurations 1 (8.59) and 2 (7.75) (Figure S2C), along with moderate improvements in median IoU scores (0.301 vs. 0.225 and 0.271) (Figure S2D). The full configuration 4, which integrated the dynamic upsampling operator, further enhanced overall performance by most closely matching the neuron count (*n* = 739 vs. *n* = 180) (Figure S2B), achieving the highest median IoU score (0.554), and obtaining the highest median PCC (0.803) between input and translated neuronal signals (Figure S2E). These quantitative metrics support our qualitative observations that, while each individual component contributed to improved performance, the integration of these components in configuration 4 was essential for the best preservation of spatial and quantitative information during translation.

### Performance evaluation of STABLE on virtual staining of fluorescence images to histological staining images

To further validate STABLE on different tasks, we conducted a virtual staining experiment by training STABLE to translate two-photon microscopy fluorescence images into H&E-stained images. The training dataset comprised fluorescence and H&E-stained images of tissue sections from head and neck tumors, representing various tissue types, such as epithelium, stroma, muscle, and glandular tissues. The training dataset consisted of image samples of 512 (*x*) × 512 (*y*) pixels from each image domain. No ground-truth paired images exist in this dataset (see the STAR Methods).

**Figure 3:**
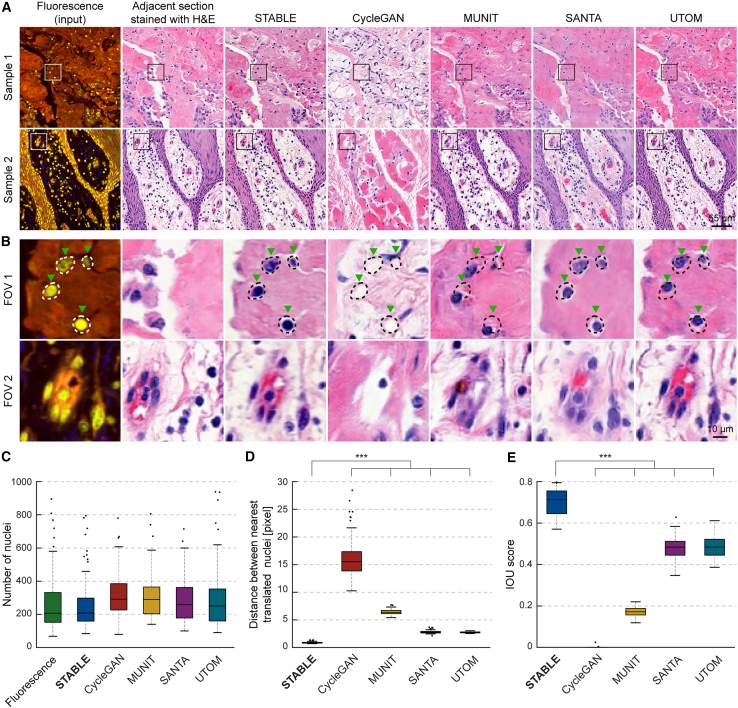
Virtual H&E staining of fluorescence microscopy data using STABLE (A) STABLE compared with unpaired image-to-image translation methods in virtual H&E staining task, converting two-photon fluorescence microscopy images to H&E-stained images. From left to right: input fluorescence images, proximate section stained with H&E, and virtual H&E staining images from STABLE, CycleGAN, MUNIT, SANTA, and UTOM. Scale bar, 55 μm. (B) Magnified views of the boxed regions from (A), focusing on regions in samples 1 and 2. Scale bar, 10 μm. (C–E) Box-and-whisker plots comparing nuclei ROI correspondence between input fluorescence and translated H&E images across STABLE and baseline methods. Nuclei ROI segmentations were obtained using Cellpose segmentation models applied to input and translated images. (C) Number of nuclei ROIs. (D) Distance between the nearest input and translated nuclei. (E) Intersection over union (IoU) scores between input and translated ROIs. Two-sample t tests were performed (*n* = 72 for each test, representing the number of test samples; n.s. *p* > 0.05, ∗*p* ≤ 0.05, ∗∗*p* < 0.01, and ∗∗∗*p* < 0.001).

We first qualitatively compared the image translation results of STABLE against the baseline methods, CycleGAN, MUNIT, SANTA, and UTOM, alongside a proximate section stained with H&E for reference (Figure 3A). The translation results of STABLE most closely resembled those of the reference H&E-stained slices, while CycleGAN, MUNIT, and SANTA produced dissimilar results. CycleGAN inverted the white background with the purple/pink foreground, while MUNIT and SANTA did not resemble the proximate stained slices in terms of hue and structure. Next, we compared the spatial accuracy of each method by analyzing close-up fields of view (FOVs) of cellular nuclei (Figure 3B). We highlighted the boundaries of the nuclei from the input image and overlaid them onto the translations (green arrows). STABLE’s results demonstrated the most accurate spatial alignment with the boundaries of the input cells, whereas the other methods either failed to translate the cells entirely (CycleGAN) or introduced substantial spatial inaccuracies (MUNIT, SANTA, and UTOM).

To quantitively assess the spatial preservation of biological structures of STABLE, we conducted a segmentation ROI-based analysis similar to the calcium imaging experiment (Figures 2B–2F) by comparing the nuclear segmentation ROIs between the input fluorescence and the translated H&E-stained images. We evaluated STABLE against baseline methods by applying similarity metrics to the segmentation ROIs. Our results confirm that STABLE outperforms existing methods in preserving precise nuclear structure. The median number of detected nuclei (208) in STABLE-generated images closely matched that of the input fluorescence images (207), while baseline methods exhibited significant deviation (CycleGAN: 291, MUNIT: 290, SANTA: 258, and UTOM: 250). The median distance between corresponding nuclei was lowest for STABLE (0.866 pixels) compared to the baseline approaches (CycleGAN: 15.5, MUNIT: 6.37, SANTA: 2.75, and UTOM: 2.72), indicating superior spatial alignment. STABLE also achieved the highest IoU score (0.713), reflecting a closer match in nuclear morphology and positioning relative to the input images compared to the lower IoU scores of CycleGAN (0.000), MUNIT (0.172), SANTA (0.484), and UTOM (0.484) (Figures 3C–3E).

### STABLE enables precisely aligned unpaired translation of DIC to DAPI fluorescence

Next, we evaluated STABLE on a virtual fluorescence labeling task by translating differential interference contrast (DIC) microscopy^40^ images into DAPI-stained^41^ fluorescence images from fixed samples derived from human breast cancer cell lines, specifically focusing on nuclei labeling.^11^ Virtual labeling offers an alternative to fluorescence labeling by reducing the reliance on costly reagents and labor-intensive staining steps, thereby accelerating research and analysis. Unlike the previous datasets, this public dataset contained ground-truth paired data. To ensure the unpaired translation paradigm and eliminate possible data leakage, we divided the dataset into two spatially non-overlapping sets. We then randomly extracted 256 (*x*) × 256 (*y*) pixel patches from the first set to form the DIC dataset while independently extracting random patches of identical dimensions from the second set for the DAPI images (see the STAR Methods).

**Figure 4:**
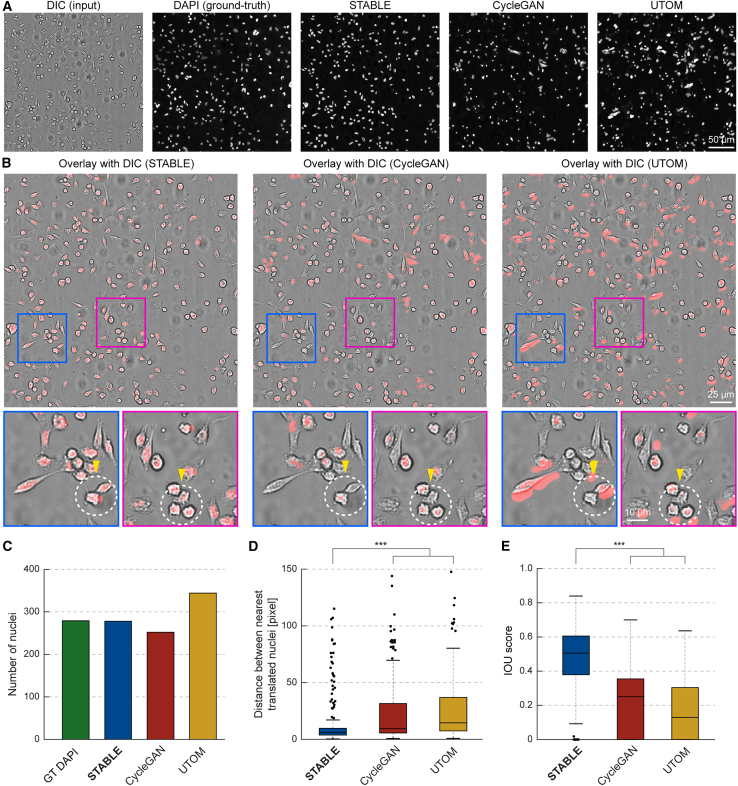
Quantitative and qualitative comparison of our method with CycleGAN and UTOM in translating DIC images to DAPI fluorescence labels (A) STABLE compared with unpaired image-to-image translation methods in translating DIC images to DAPI fluorescence labels. From left to right: input DIC image, corresponding ground-truth (GT) DAPI fluorescence image, and translated DAPI images from STABLE, CycleGAN, and UTOM. Scale bar, 50 μm. (B) Overlay of the translated fluorescence signals (red) on the original DIC (gray) images for each method. Magnified views of the highlighted regions (pink and blue boxes). Scale bar for large views, 25 μm. Scale bar for magnified views, 10 μm. (C–E) Box-and-whisker plots comparing nuclei ROI correspondence between GT and translated DAPI images across STABLE and baseline methods. Nuclei ROI segmentations were obtained using a Cellpose segmentation model applied to the GT and translated DAPI images. (C) Number of nuclei ROIs. (D) Distance between the nearest input and translated nuclei. (E) Intersection over union (IoU) scores between input and translated ROIs. Two-sample t tests were performed (*n* = 279 for each test, representing the number of GT DAPI nuclei; n.s. *p* > 0.05, ∗*p* ≤ 0.05, ∗∗*p* < 0.01, and ∗∗∗*p* < 0.001).

To evaluate our method’s performance, we compared STABLE against two baseline methods: CycleGAN and UTOM. Qualitatively, the DAPI images produced by our method exhibit a closer resemblance to the ground truth compared to those generated by CycleGAN and UTOM (Figure 4A). When overlaying the predicted DAPI images onto the input DIC images, our method demonstrated superior alignment, with the DAPI labels well positioned relative to the DIC features (Figure 4B). In contrast, the CycleGAN and UTOM models occasionally produced misaligned or incorrectly positioned nuclei, leading to inaccurate results.

We extended the segmentation-based quantitative analysis by comparing the nuclear segmentation ROIs from the ground-truth DAPI data with the translated DAPI images. These results confirm that STABLE-generated DAPI images exhibit nuclear counts (279) that most closely match those of the ground-truth DAPI images (278) compared to the baseline methods (CycleGAN: 252 and UTOM: 344). Furthermore, STABLE achieves the smallest median centroid distance (6.03) compared to CycleGAN (9.44) and UTOM (14.6). With respect to spatial overlap metrics, STABLE attains the highest median IoU score (0.505), exceeding those of both CycleGAN (0.251) and UTOM (0.129) baselines (Figures 4C–4E).

### STABLE enables accurate and artifact-free unpaired translation from T2- to PD-weighted MRI

We moved to a non-optical image translation task of converting T2 (transverse relaxation time)-weighted MRI data to PD (proton density)-weighted MRI data using a public dataset of volumetric images from normal, healthy subjects.^42^ T2-weighted MRI highlights tissue differences based on transverse relaxation times, making water-rich structures appear bright, which is useful for tracking conditions such as edema, tumors, and inflammation. On the other hand, PD-weighted MRI emphasizes the density of hydrogen protons within tissues, making them ideal for examining anatomical structures like the brain and musculoskeletal tissues.^43^ Converting T2 images into PD images could enable more comprehensive clinical diagnoses in cases where PD images are unavailable, such as in the diagnosis of multiple sclerosis (MS), where both inflammation (T2) and structural changes (PD) are crucial for a thorough evaluation.^44^ This is particularly important in cases where acquiring both T2 and PD images may be challenging or uncomfortable for patients.

The dataset contained paired MRI scans from the same human subjects across different modalities (T2 and PD). To remove any ground-truth pairs, we divided the dataset by randomly assigning different human subjects to each modality group, where half of the subjects were allocated to the T2 group and the remaining half to the PD group. This separation prevented ground-truth pairs from being included in our training data, as no subject appeared in both modality groups simultaneously. For the training data, we then randomly sampled 1 × 256 × 256 z-slices from the full volumetric MRI scans (see the STAR Methods).

**Figure 5:**
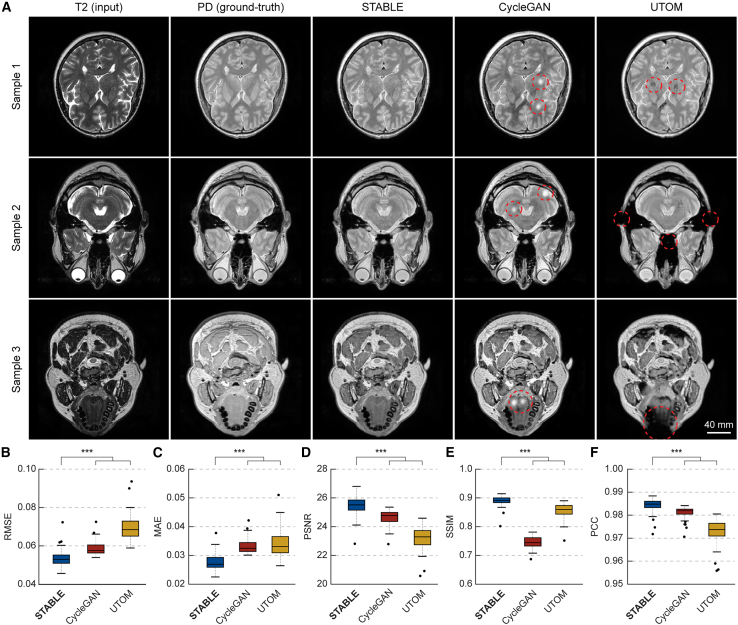
Virtual MRI modality transfer using STABLE (A) STABLE compared with unpaired image-to-image translation methods in translating T2-weighted MRI to PD-weighted MRI data. From left to right: input T2-weighted MRI (left), corresponding GT PD-weighted MRI (second column), followed by the results from STABLE, CycleGAN, and UTOM. Example slices from three different subjects are presented. Red circles highlight areas where CycleGAN and UTOM generate artifacts or fail to capture fine structural details. Scale bar, 44 mm. (B–F) Box-and-whisker plots showing the quantitative comparison between methods across several evaluation metrics: (B) RMSE, (C) MAE, (D) PSNR, (E) SSIM, and (F) PCC. Two-sample t tests were performed (*n* = 64 for each test, representing the number of volumetric MRI samples; n.s. *p* > 0.05, ∗*p* ≤ 0.05, ∗∗*p* < 0.01, and ∗∗∗*p* < 0.001).

We compared the performance of our method with those of two baseline approaches, CycleGAN and UTOM. Qualitatively, our method demonstrated superior fidelity to ground-truth PD images (Figure 5A; Video S2). The images produced by our method exhibited minimal artifacts, whereas CycleGAN and UTOM frequently generated visible white spots or other artifacts.

Video S2. MRI modality data translation, related to Figure 5

Quantitative evaluation was conducted using multiple similarity metrics between the ground-truth PD and the translated PD MRI data, including root-mean-squared error (RMSE), mean absolute error (MAE), peak signal-to-noise ratio (PSNR), structural similarity index measure (SSIM), and PCC. Our method consistently outperformed both CycleGAN and UTOM across all metrics. Specifically, STABLE achieved superior results, with a median RMSE of 0.0530, MAE of 0.0269, PSNR of 25.5, SSIM of 0.891, and PCC of 0.985. By comparison, CycleGAN yielded an RMSE of 0.0577, MAE of 0.0325, PSNR of 24.8, SSIM of 0.744, and PCC of 0.982, while UTOM produced an RMSE of 0.0685, MAE of 0.0331, PSNR of 23.3, SSIM of 0.860, and PCC of 0.974 (Figures 5B–5F).

### STABLE enables unpaired translation of high-SNR images from low-SNR images

An additional study was conducted to see if STABLE could be used to recover fine morphological details from low-SNR fluorescence images while maintaining structural details by translating noisy low-SNR images to high-SNR ones. We evaluated STABLE on two public volumetric microscopy SNR reconstruction datasets: penicillium^45^ and planaria.^16^

These datasets contain paired low-SNR and high-SNR 3D volumes. Therefore, to prevent ground-truth correspondence, for the penicillium dataset, we divided the full volumetric data into two non-overlapping sets, using one half for the high-SNR dataset and the other half for the low-SNR dataset. We then generated the training dataset by randomly sampling and cropping the volumetric data into 256 (*x*) × 256 (*y*) patches within their respective partitions. For the planaria dataset, which was already sampled into paired 16 (*z*) × 64 (*x*) × 64 (*y*) volumes, we eliminated ground-truth pairs by splitting the collection of volumes so that half of the volumetric patches were assigned to the high-SNR dataset and the remaining half to the low-SNR dataset. For training, we randomly sampled a single 1 (*z*) × 64 (*x*) × 64 (*y*) pixel z-slice from each of the training volumes (see the STAR Methods).

Our results demonstrate that STABLE can be used to restore the low-SNR fluorescence images in both datasets. Figure S3A qualitatively shows that STABLE-generated high-SNR penicillium data provide better contrast, with previously obscured details becoming visible. We further verify the results through line profiles of normalized intensity across selected regions (Figure S3B). Line profiles of normalized intensity across selected regions confirm that denoised images from STABLE better align with ground-truth high-SNR data, with noise components reduced. To quantitatively support our results, we compute and compare key similarity metrics (RMSE, MAE, PSNR, SSIM, and PCC) between the low-SNR images with the ground-truth high-SNR images and the restored images using STABLE with the ground-truth high-SNR images. We can see a significant improvement across all metrics. When comparing against ground-truth high-SNR images, STABLE demonstrated a significant reduction in median RMSE (0.0858 vs. 0.134), lower median MAE (0.0593 vs. 0.0941), higher median PSNR (30.5 vs. 26.8), higher median SSIM (0.734 vs. 0.541), and higher median PCC (0.861 vs. 0.635) than the original low-SNR images (Figures S3C–S3G).

The translation results from the planaria dataset showed consistent results (Figure S4): lower median RMSE (0.0510 vs. 0.201), lower median MAE (0.0360 vs. 0.176), higher median PSNR (35.89 vs. 25.36), higher median SSIM (0.762 vs. 0.232), and higher median PCC (0.844 vs. 0.398).

## Discussion

In this study, we proposed STABLE to address the challenges of preserving accurate spatial and quantitative information in unpaired image-to-image translation for biomedical images. Unlike existing models that rely solely on cycle consistency constraints, STABLE introduces information consistency and dynamic upsampling operators to allow more accurate translations.

The significance of both information consistency and dynamic upsampling in STABLE was demonstrated through our experimental results. The introduction of information consistency allowed the model to preserve information in the feature space, mitigating the limitations of image cycle consistency when modeling complex translation relationships. This is supported by the calcium imaging translation results: the baseline methods incorrectly translated the nuclear-localized neurons to the edges and intersections of the input cytosolic neurons. This misplacement was likely due to an over-reliance on cycle consistency, where the models learned translation relationships that prioritized pixel-wise consistency, thereby neglecting the true spatial locations and shapes of the neurons by placing the high-intensity nucleus-localized neurons at the bright edges of the input cytosolic neurons. In contrast, STABLE’s information consistency terms reduced the dependency on the cycle consistency loss, allowing the model to learn important spatial features. Furthermore, STABLE’s incorporation of dynamic upsampling operators was essential for improving spatial accuracy, thus addressing the misalignment issues present in methods using conventional upsampling operators.

It is theoretically possible to use alternative operators that learn to upsample features in STABLE. To see the effect of alternative dynamic upsampling operators, we implemented a simple learnable operator using deformable convolution,^46^ where we first upsampled the feature map using fixed bilinear interpolation and then applied a deformable convolution layer. We compared the results obtained from this deformable convolution-based implementation with the original STABLE implementation on the calcium imaging dataset. Both approaches performed comparably in terms of preserving spatial information in the calcium imaging translation task (Figures S2F–S2J). This demonstrates that the integration of the concept of dynamic upsampling with image translation, rather than the specific implementation, is key to STABLE’s performance.

### Limitations of the study

While STABLE demonstrates superior performance in preserving spatial and quantitative features in unpaired image translation, it is crucial to acknowledge its limitations. The translation network, whether trained with paired or unpaired data, operates within the confines of the information present in the input image. It cannot generate or introduce new information that is not already encoded within the source data. Therefore, while STABLE is designed to preserve spatial and quantitative information, the quality of translation is inherently dependent on the underlying dataset. If the two modalities share a significant amount of structural and intensity-related features, the translation process is more reliable. Conversely, when the two modalities have minimal overlap in structural or semantic information, translation performance degrades, which can result in hallucinated or unrealistic outputs.

Specifically, certain structures in the output may be affected by ambiguous or incomplete information in the input when shared data are limited, such as in the case of imaging artifacts. For instance, false nuclei may be generated in the DAPI image due to ambiguous structures in the input DIC image (Figure S5A). Furthermore, intensity differences can arise from domain shifts in the dataset caused by sample-to-sample differences in preparation or imaging modalities, as shown in the slight difference in overall intensity between the translated PD image and the ground truth (Figure S5B). This may be because the model learns to generate outputs that conform to the learned target domain distribution, which may not exactly match the specific intensity characteristics of individual input samples.

To illustrate this point in a more empirical manner, we have conducted a study that systematically investigates how translation performance deteriorates as the amount of shared information decreases. Here, we specifically analyze the impact of reducing both the dataset size and, therefore, the amount of shared information. We used the calcium imaging dataset and progressively reduced the training data from 100% to 75%, 50%, 25%, 10%, and 5% of the original dataset while keeping the test set constant. The results reveal a clear degradation pattern as shared data are reduced: increased hallucinations where neurons appear in incorrect anatomical locations, a significant reduction in the overall number of detected neurons, and diminished accuracy in structural feature preservation (Figure S5C).

Given this constraint, it becomes both necessary and critically important to rigorously validate the network for each specific translation task. The validation process should encompass key aspects such as accuracy of feature preservation, consistency across diverse datasets, absence of artificial artifacts, comparison with ground truth when possible, and expert evaluation of biological plausibility.

## Resource availability

### Lead contact

Requests for further information and resources should be directed to and will be fulfilled by the lead contact, Dr. Young-Gyu Yoon (ygyoon@kaist.ac.kr).

### Materials availability

Fish lines are available through the lead contact upon reasonable request.

The recombinant DNA constructs generated in this study are not available as physical plasmids. The corresponding DNA sequences are available from the lead contact upon reasonable request.

### Data and code availability

• Calcium imaging data have been deposited at Figshare and are publicly available as of the date of publication at https://doi.org/10.6084/m9.figshare.28707218*.* Virtual H&E staining data have been deposited at Figshare and are publicly available as of the date of publication at https://doi.org/10.6084/m9.figshare.28707146*.*
• All original code has been deposited at Figshare and is publicly available at https://doi.org/10.6084/m9.figshare.28936559 as of the date of publication.
• Any additional information required to reanalyze the data reported in this work paper is available from the lead contact upon request.

## Acknowledgments

The zebrafish lines used for calcium imaging were provided by the Zebrafish Center for Disease Modeling, Korea. This research was supported by the 10.13039/501100003725National Research Foundation of Korea (NRF) (RS-2023-00209473, RS-2023-00264409, and RS-2024-00349650); a 10.13039/501100003716Korea Basic Science Institute (National Research Facilities and Equipment Center) grant funded by the 10.13039/501100014188Ministry of Science and ICT (RS-2024-00401676); the Bio & Medical Technology Development Program through the NRF, funded by the Ministry of Science and ICT (RS-2021-NR056586); the BK21 Plus Program through the NRF, funded by the Ministry of Education of Korea; and 10.13039/100019266Korea Medical Device Development Fund grants funded by the Korean government (1711137947 and KMDF_PR_20200901_0027).

## Author contributions

J.Y.Y. designed and implemented the proposed method. J.Y.Y. performed the image translation experiments with M.E., M.L., and C.S. J.Y.Y. and M.E. performed the analysis of the experimentational results. J.Y.Y. and M.E. performed denoising of the data. T.-I.C. performed transgenic zebrafish experiments under the supervision of C.-H.K. J.Y.Y., M.E., and E.-S.C. performed confocal imaging of zebrafish. J.C., J.M., and E.K. performed two-photon fluorescence imaging and histology imaging of tissue under the supervision of P.K. J.Y.Y., M.E., and Y.-G.Y. wrote the manuscript with input from all authors. Y.-G.Y. conceived and led this work.

## Declaration of interests

The authors declare no competing interests.

## Declaration of generative AI and AI-assisted technologies in the writing process

During the preparation of this work, the authors used ChatGPT as a tool to correct the grammatical mistakes. After using this tool, the authors reviewed and edited the content as needed and take full responsibility for the content of the publication.

## STAR★Methods

### Key resources table

**Table:** 

REAGENT or RESOURCE	SOURCE	IDENTIFIER
**Biological samples**		

Resected tissue of patients with head and neck cancer tumors	Seoul National University Bundang Hospital	IRB No. B-2103-673-312

**Deposited data**		

Calcium Imaging Dataset	This paper	https://doi.org/10.6084/m9.figshare.28707218
Virtual H&E Staining Dataset	This paper	https://doi.org/10.6084/m9.figshare.28707146

**Experimental models: Organisms/strains**		

Tg(HuC: Gal4, UAS: GCaMP7a)	Zebrafish Center for Disease Modeling, ZCDM	N/A
Tg(HuC: Gal4, UAS: RiboL1-jGCaMP8m)	This paper	N/A
Tg(HuC: H2B-GCaMP6s)	This paper	N/A

**Recombinant DNA**		

pTol2-UAS-RiboL1-jGCaMP8m	This paper	N/A

**Software and algorithms**		

STABLE	This paper	https://doi.org/10.6084/m9.figshare.28936559
MATLAB	Mathworks	https://www.mathworks.com/products/matlab.html
Python	Python	https://www.python.org/
Pytorch	Meta AI	https://pytorch.org/
Numpy	Numpy	https://numpy.org/
scikit-image	scikit-image	https://scikit-image.org/
CUDA	Nvidia Corporation	https://developer.nvidia.com/cuda-toolkit
SUPPORT	Eom et al.^45^	https://github.com/NICALab/SUPPORT
DySample	Liu et al.^31^	https://github.com/tiny-smart/dysample
Cellpose	Stringer et al.^39^	https://github.com/MouseLand/cellpose

### Experimental model and study participant details

#### Zebrafish husbandry

All animal experiments involving zebrafish (*Danio rerio*) conducted for this study were approved by the IACUC of KAIST (KA-2021-125). Zebrafish were kept at a 10h dark, 14h light cycle at 28C. Larval sex is not specified at this developmental stage and was therefore not determined.

#### H&E histology samples

A retrospective case study was conducted to collect unstained formalin-fixed paraffin-embedded (FFPE) slides and Hematoxylin and Eosin (H&E) stained slides from archived tissue samples of patients diagnosed and treated for head and neck cancer at Seoul National University Bundang Hospital (SNUBH). The samples were retrieved from the hospital’s pathology archives and included tissue from patients diagnosed before February 2013. This study was approved by the Institutional Review Board of SNUBH (IRB No. B-2013-673-312). The requirement for informed consent was waived. All patient data were strictly anonymized using non-identifiable codes to ensure confidentiality.

### Method details

#### STABLE network training pipeline and loss functions

Given images $X_{A}\in R^{C_{A}\times H\times W}$ and $X_{B}\in R^{C_{B}\times H\times W}$, randomly sampled from their respective image domains A and B, the goal of STABLE is to learn two translation functions, $T_{A\to B}$ and $T_{B\to A}$, that map images between the domains while preserving key semantic information (Figure S1A). These functions are modeled as conditional generators, where:

$$X_{A\to B}=T_{A\to B}\left(X_{A}\right)\;\text{and}\;X_{B\to A}=T_{B\to A}\left(X_{B}\right)\text{.}$$

$X_{A\to B}\in R^{C_{B}\times H\times W}$ and $X_{B\to A}\in R^{C_{A}\times H\times W}$ correspond to the translated images, where $X_{A\to B}$ is the domain B image translated from domain A and vice versa. Discriminators $D_{A}$ and $D_{B}$, modeled as neural network-based classifiers, are trained adversarially against the generators. The adversarial loss encourages the generators to produce images indistinguishable from real images in the target domain.^47^ The adversarial loss function is defined as:

$$L_{adv}\left(T_{A\to B},T_{B\to A},D_{A},D_{B}\right)=E_{X_{A}}\left[\log\;D_{A}\left(X_{A}\right)\right]+E_{X_{A}}\left[\log\left(1-D_{B}\left(T_{A\to B}\left(X_{A}\right)\right)\right)\right]+{\;E}_{X_{B}}\left[\log\;D_{B}\left(X_{B}\right)\right]+E_{X_{B}}\left[\log\left(1-D_{A}\left(T_{B\to A}\left(X_{B}\right)\right)\right)\right]\text{.}$$

Since paired ground-truth data is unavailable, the cycle consistency loss is introduced to allow the translation retains sufficient information to reconstruct the original input image. The cycle consistency loss is defined as:

$$L_{cyc}\left(T_{A\to B},T_{B\to A},D_{A},D_{B}\right)={\left|X_{A}-T_{B\to A}\left(T_{A\to B}\left(X_{A}\right)\right)\right|}_{1}+{\left|X_{B}-T_{A\to B}\left(T_{B\to A}\left(X_{B}\right)\right)\right|}_{1}\text{.}$$

Despite this, the image cycle consistency loss alone does not guarantee accurate translations. STABLE addresses this by introducing an information consistency loss to constrain the spatial features encoded from the input and translated images. To implement this, the generators $T_{A\to B}$ and $T_{B\to A}$ are implemented as encoder-decoder pairs:

$$T_{A\to B}=F_{B}\left(G_{A}\left(X_{A}\right)\right),T_{B\to A}=F_{A}\left(G_{B}\left(X_{B}\right)\right)\text{,}$$

where the encoders $G_{A}$ and $G_{B}$ extract feature maps of equal spatial dimensions as the input images: $Z_{A}\in R^{C_{Z}\times H\times W}$ and $Z_{B}\in R^{C_{Z}\times H\times W}$ from input images $X_{A}$ and $X_{B}$, respectively, i.e., $Z_{A}=G_{A}\left(X_{A}\right)$ and $Z_{B}=G_{B}\left(X_{B}\right)$. The decoders $F_{A}$ and $F_{B}$ then decode the features maps into their corresponding domain images: $X_{A\to B}=F_{B}\left(Z_{A}\right)$ and $X_{B\to A}=F_{A}\left(Z_{B}\right)$.

The information consistency loss minimizes the difference between the feature maps encoded from the input and translated images:

$$L_{info}\left(G_{A},G_{B},F_{A},F_{B}\right)={\left|Z_{A}-G_{B}\left(X_{A\to B}\right)\right|}_{1}+{\left|Z_{B}-G_{A}\left(X_{B\to A}\right)\right|}_{1}\text{.}$$

The total loss function is a weighted sum of the adversarial loss, cycle consistency loss, and information consistency loss:

$$L_{tot}=\lambda_{adv}L_{adv}+\lambda_{cyc}L_{cyc}+\lambda_{info}L_{info}\text{.}$$

#### STABLE network architecture

The architecture of the STABLE network consisted of two encoders, two decoders, and two discriminators, each associated with one image domain (Figure S1A).

The encoders and decoders were implemented as custom U-shaped convolutional networks^48^ (Figure S1C). Each network was comprised of a contracting path and an expansive path connected via skip connections that merged feature maps from the contracting path with their corresponding feature maps in the expansive path. The contracting path was composed of convolutional blocks, where each block contained two 3 × 3 convolution layers, each followed by a ReLU activation function. After each block, a 2 × 2 max pooling layer was used to reduce the spatial resolution. In the expansive path, the spatial resolution of the feature maps was doubled using the dynamic upsampling operator and concatenated with those from the contracting path before being processed by two additional 3 × 3 convolution layers, each followed by a ReLU activation function.

Specific architectural designs were implemented to prevent feature misalignments and artifacts that degraded the translation quality. First, the traditional fixed bilinear upsampling operators were replaced with learnable, dynamic upsampling operators^31^ (Figure S1D), which improved spatial alignment during translation (Figure 1C). The dynamic upsampling operator predicts an offset sampling grid that adjusts sampling locations dynamically, ensuring that each upsampled pixel is extracted from an optimal location in the low-resolution feature map that minimizes the training loss.

The dynamic upsampling operator learns to upsample the feature maps by learning an offset grid that is used to resample the feature map to a higher resolution. This resampling process, also known as grid sampling, is a process in which an output feature map is constructed by sampling values from an input feature map at specific positions. This allows transformations such as warping, scaling, and rotation to be applied to images in a differentiable manner. When applied to upsampling, grid sampling enables an input feature map to be resampled at a higher resolution by specifying a grid of sampling coordinates that dictate where the output values should be extracted from the input.

To formulate this sampling process, assume a feature map X of dimensions C×H×W. We want to scale this feature map by a scale factor of s to obtain an upsampled feature map $X^{\prime }$ of dimensions C×sH×sW. We define a sampling grid G as a 2-channel tensor of dimension 2×sH×sW, where s is the scaling factor. This sampling grid contains the x and y coordinates from which we will sample the original feature map X to obtain the upsampled feature map X’, where one of the two channels contains the x coordinates and the other contains the y coordinates. We can then formulate the grid sampling process for pixel at coordinates (u,v) of the upsampled feature map X’ as:

$$X^{\prime }\left(u,v\right)=X\left(G_{x}\left(u,v\right),G_{y}\left(u,v\right)\right)\text{,}$$

where $G_{x}\left(u,v\right)$ and $G_{y}\left(u,v\right)$ correspond to the x and y coordinates from where we will resample the pixel value from X. However, since the sampling locations $G_{x}\left(u,v\right)$ and $G_{y}\left(u,v\right)$ might not necessarily align with discrete pixel positions in X, the values at these intermediate positions are obtained via interpolation.

In fixed upsampling, the sampling grid will contain fixed and uniformly distributed coordinates. This fixed approach assumes that every upsampled pixel should be extracted from predefined locations, which does not account for potential spatial misalignments or distortions that may arise in biomedical image translation tasks. To overcome this limitation, the dynamic upsampling operator does not simply sample at these fixed positions. Instead, it learns an offset grid O(u,v)=(Δx(u,v),Δy(u,v)), which is added to the standard sampling grid G to dynamically adjust the sampling locations based on the content of X. This offset map is computed by:

$$O_{\theta }\left(u,v\right)=Conv2D\left(X;\theta \right)\text{,}$$

where Conv2D(·) is a 2D convolutional layer with a 1×1 kernel applied to the input feature map X, and θ represents its learnable parameters. This operation generates an output of dimension $2s^{2}\times H\times W$. Since this offset tensor is initially computed at the low-resolution scale, it must be restructured to match the upsampled grid. This is accomplished using pixel shuffle,^32^ an operation that rearranges values from the channel dimension into the spatial dimensions, effectively increasing the spatial resolution of the offset grid. This transformation reshapes the tensor to the upsampled spatial resolution of 2×sH×sW, ensuring that each upsampled pixel has a corresponding learned displacement vector.

The final sampling grid $S_{\theta }$ is then obtained by adding the learned offset $O_{\theta }$ to the standard grid G:

$$S_{\theta }\left(u,v\right)=G\left(u,v\right)+O_{\theta }\left(u,v\right)\text{.}$$

Using the previously defined grid sampling operation, we can upsample the input feature map X to the target resolution of C×sH×sW using the learned sampling positions $S_{\theta }$.

Weight modulation and demodulation^49^ were implemented to prevent droplet-like artifacts and improve the accuracy of the intermediate feature maps.

The discriminators were implemented as Least Squares GAN (LSGAN) discriminators.^50^ Each discriminator (Figure S1E) was comprised of a sequence of 4 × 4 convolutional layers, followed by batch normalization^51^ and a LeakyReLU activation function. The final layer was a 3 × 3 convolution that output a single-channel feature map for computing the least squares loss.

#### Training details and parameters

For all experiments, the network was trained using Python 3.11.9, Pytorch 2.3.0,and CUDA 12.4 on an NVIDIA RTX 4090 GPU and an Intel Xeon Silver 4410T CPU. A batch size of 1 was used across all experiments. All loss weight values and the number of feature map channels were chosen based on a standard hyperparameter sweep by varying selected parameters. The loss weights were swept from a range of 1, 2, 5, 10 and the channels were swept from 1, 3, and 8. We include the results from a loss weight sensitivity test conducted on a subset of the calcium imaging dataset in Supplementary Table S34 and the results from a feature map channel number sensitivity test in Supplementary Table S35. All networks in STABLE including the U-Nets are trained in an end-to-end fashion without using any external or pretrained weights. The optimization was performed using the AdamW optimizer^52^ with a learning rate of $3\times 10^{-4}$ and no weight decay.

#### Training and dataset details for image translation on calcium imaging data

For the calcium imaging experiment, the raw images were denoised using the SUPPORT^45^ algorithm. The full dataset included calcium imaging recordings from five cytosolic and six nuclear-localized zebrafish samples. The cytosolic and nuclear-localized samples are from different animals; therefore, there are no ground-truth pairs in this dataset.

The cytosolic recordings were acquired at 0.58 Hz with a pixel size of 0.45 μm, where four are of dimensions 240 (t) × 512 (x) × 512 (y) and one is of 290 (t) × 1024 (x) × 1024 (y). The nuclear-localized samples were images at 0.5 Hz with a pixel size of 0.34 μm, where 23 260 (t) × 512 (x) × 512 (y) recordings were acquired. For training, we use four of the cytosolic recordings, while we keep one unseen during training for evaluation.

For data preprocessing, the images were normalized by scaling with the 99 percentile value. We then randomly sampled frames from the recordings and randomly cropped them into 1 (t) × 256 (x) × 256 (y) pixel patches. Data augmentation involved random rotations of 90° and random vertical and horizontal flips. The dimensions of the data used in training were 1 (t) × 256 (x) × 256 (y) pixels.

The loss weights used for this experiment were $\lambda_{adv}=1$, $\lambda_{info}=10$, and $\lambda_{cyc}=5$. In this task, the cycle consistency loss weight was increased following a sigmoid growth function during training for improved stability. The network architecture consisted of three spatial levels with channel sizes of 8, 16, and 32. The intermediate feature map Z, used for information consistency, had 8 channels.

Calcium imaging data exhibits a uniquely broad dynamic range with both extremely high and low intensity values present within a single sample. This intra-sample variability in intensity means that batch normalization, which applies global rescaling and shifting, can distort the natural intensity distribution and degrade performance. In contrast, other imaging modalities have more uniform intensity profiles and tolerate batch normalization well. Consequently, we omitted batch normalization for the calcium imaging experiment.

#### Training and dataset details for image translation on virtual H&E staining data

For the H&E virtual staining experiment, the full dataset comprised of fluorescence images and H&E-stained images of tissue sections from head and neck tumors, representing various tissue types such as epithelium, stroma, muscle, and glandular tissues. The dataset included a total of 512 image patches per domain, with each patch having dimensions of 512 (x) × 512 (y) pixels and a pixel size of 0.55 μm. No ground-truth paired images exist in this dataset. The dataset was split into 440 training samples and 72 test samples for evaluation.

For data preprocessing, the images were normalized to a range of 0–1 and randomly shuffled. Data augmentation was performed by applying random rotations of 90° and randomly flipping the images both vertically and horizontally. All training images retained their original dimensions of 512 (x) × 512 (y) pixels.

The loss weights for the H&E virtual staining experiment were $\lambda_{adv}=5$, $\lambda_{info}=10$, and $\lambda_{cyc}=10$. The encoder and decoder networks for this task included four spatial levels with channel sizes of 32, 64, 128, and 256. The intermediate feature map Z consisted of three channels. Batch normalization was used.

#### Training and dataset details for image translation on DAPI virtual fluorescent labeling data

For the DAPI virtual labeling experiment, we used a public dataset available at https://github.com/google/in-silico-labeling?tab=readme-ov-file.^11^ This dataset includes volumetric Differential Interference Contrast (DIC) microscopy images and DAPI-stained fluorescence images. The dataset consisted of two paired samples of volumetric DIC data of 13 (z) × 3470 (x) × 3470 (y) pixels and corresponding DAPI label images of 3470 (x) × 3470 (y) pixels with a pixel size of 6.5 μm. We used one sample for training and reserved the other for evaluation.

To eliminate ground-truth pairs and possible data leakage, we divided the dataset into two spatially non-overlapping sets, each corresponding to either the DIC or DAPI dataset. From the first set, we randomly extracted 1 (z) × 256 (x) × 256 (y) pixel patches to form the DIC dataset, while independently extracting random patches of identical dimensions from the second set for the DAPI dataset.

For data preprocessing, the images were first normalized by the 0.1 and 99.0 percentiles, and data augmentation was performed by applying random rotations of 90°, and randomly flipping the images both vertically and horizontally. The dimensions of the data used in training were 256 (x) × 256 (y) pixels.

The loss weights for the virtual DAPI labeling experiment were $\lambda_{adv}=1$, $\lambda_{info}=5$, and $\lambda_{cyc}=1$ 0. The encoder and decoder networks for this task included four spatial levels with channel sizes of 32, 64, 128, and 256. The intermediate feature map Z consisted of 8 channels. Batch normalization was used.

#### Training and dataset details for image translation on MRI modality transfer data

For the MRI modality transfer experiment, we used a public dataset available at https://brain-development.org/ixi-dataset.^42^ This dataset includes 318 paired T2-weighted and PD-weighted volumetric MRI samples. Each volume is of 100 (z) × 256 (x) × 256 (y) voxels. We used 254 samples for training and 64 for evaluation.

The T2-weighted images were acquired with the following settings: Repetition time = 8178.34, Echo time = 100, Number of Phase Encoding Steps = 187, Echo Train Length = 16, Reconstruction Diameter = 240, and Flip Angle = 90. The PD images were acquired with: Repetition time = 8178.34, Echo time = 8, Number of Phase Encoding Steps = 187, Echo Train Length = 16, Reconstruction Diameter = 240, and Flip Angle = 90.

To remove ground-truth pairs, we divided the full dataset by selecting half of the human subjects for the T2 dataset and the other half for the PD dataset.

For data preprocessing, the images were first normalized by the 0.1 and 99.9 percentiles. We randomly sampled a z-slice from each volume and then performed data augmentation by applying random rotations of 90°, and flipping the images both vertically and horizontally. The dimensions of the data used in training were 1 (z) × 256 (x) × 256 (y) pixels.

The loss weights for the MRI modality transfer experiment were $\lambda_{adv}=1$, $\lambda_{info}=1$, and $\lambda_{cyc}=1$ 0. The encoder and decoder networks for this task included four spatial levels with channel sizes of 32, 64, 128, and 256. The intermediate feature map Z consisted of three channels. Batch normalization was used.

#### Training and dataset details for image translation on the low SNR to high SNR penicillium data

For the low SNR to high SNR translation experiment on the penicillium data, we used a public dataset available at Zenodo Data: https://doi.org/10.5281/zenodo.8176722.^45^ This dataset includes a pair of volumetric low SNR and high SNR samples of 1000 (z) × 1024 (x) × 1024 (y) voxels. The frame rate was 0.5 Hz with a pixel size of 0.34 μm and z-step size of 0.1 μm. From the full volume, we used 900 z-slices for training and 100 slices for evaluation.

To remove ground-truth pairs, we divided the volumetric data into two spatially non-overlapping sets. We used one-half for the high SNR dataset and the other half for the low SNR dataset. We then generated the training dataset by randomly sampling and cropping the split volumetric data into 256 (x) × 256 (y) patches within their respective partitions.

For preprocessing, the images were first normalized by the 0.1 and 99.9 percentiles. Data augmentation was performed by applying random rotations of 90°, and random flipping of the images both vertically and horizontally. The dimensions of the data used in training were 1 (z) × 256 (x) × 256 (y) pixels images.

The loss weights were $\lambda_{adv}=1$, $\lambda_{info}=1$, and $\lambda_{cyc}=1$ 0. The encoder and decoder networks for this task included four spatial levels with channel sizes of 32, 64, 128, and 256. The intermediate feature map Z consisted of three channels. Batch normalization was used.

#### Training details for image translation on the low SNR to high SNR planaria data

For the low SNR to high SNR translation experiment on the planaria data, we used a public dataset available at https://publications.mpi-cbg.de/publications-sites/7207.^16^ This dataset includes 17,900 randomly sampled paired volumes of 16 (z) × 64 (x) × 64 (y) voxels for training and 20 evaluation volumes of varying dimensions: 16 samples of 95 (z) × 1024 (x) × 1024 (y), and one sample each of 38 (z) × 1024 (x) × 1024 (y), 60 (z) × 1024 (x) × 1024 (y), 26 (z) × 1024 (x) × 1024 (y), and 68 (z) × 1024 (x) × 1024 (y). The exact pixel size was not reported in the original study.

To eliminate ground-truth pairs, we split the collection of training volumes so that half of the volumetric patches were assigned to the high SNR dataset and the remaining half to the low SNR dataset.

For training, the images were first normalized by the 0.1 and 99.9 percentiles and then a single z-slice was randomly sampled from each training volume, resulting in 1 (z) × 64 (x) × 64 (y) pixel images. Data augmentation was performed by applying random rotations of 90°, and random flipping of the images both vertically and horizontally.

The loss weights were $\lambda_{adv}=1$, $\lambda_{info}=1$, and $\lambda_{cyc}=1$ 0. The encoder and decoder networks for this task included four spatial levels with channel sizes of 32, 64, 128, and 256. The intermediate feature map Z consisted of three channels. Batch normalization was used.

#### Segmentation of regions of interest

For the automatic segmentation for the ROI analysis, we used Cellpose 3^39^, publicly available at https://github.com/MouseLand/cellpose. For each dataset involving the ROI analysis, we trained separate Cellpose models on a subset of manually annotated ground-truth segmentation data to ensure that the segmentation model was appropriately calibrated for our specific imaging conditions. The manual annotation process was performed independently on each image, following a consistent protocol using the Cellpose GUI.

All Cellpose models were trained using the Cellpose 3 GUI version on Windows with the following training parameters: learning rate = 0.01, weight decay = 0.0001, number of epochs = 10,000. For the single channel image datasets (calcium imaging, DIC to DAPI), the channel to segment was set to ‘gray’ and the second channel was set to ‘none’. For the two-photon fluorescence images, the channel to segment was set to ‘green’ and the second channel was set to ‘none’. For the H&E staining images, the channel to segment was set to ‘red’ and the second channel was set to ‘none’ For segmentation, we used the following segmentation parameters: flow threshold = 0.4, cell probability threshold = 0.0, normalization percentiles = [1.0, 99.0], and niter dynamics = 0. We did not use any of the image restoration features.

Training and segmentation were performed on a workstation with one Intel Core i7-9700K CPU, 128 GiB of RAM, and an NVIDIA GeForce RTX 2070 GPU.

#### Denoising calcium imaging data

We used SUPPORT,^45^ publicly available at https://github.com/NICALab/SUPPORT, to denoise calcium imaging data. SUPPORT effectively eliminates zero-mean noise in the data by learning and utilizing the spatial and temporal dependencies among pixel values. The SUPPORT network was trained with a batch size of 64 and a patch size of 61(t) × 128(x) × 128(y). The SUPPORT network had a receptive field size of 61(t) × 146(x) × 146(y) and a blind spot size of 1(t) × 1(x) × 1(y).

Denoising was performed on a workstation with two Intel Xeon Gold 6226R CPUs, 128 GiB of RAM, and an NVIDIA GeForce RTX 4090 GPU.

#### Baseline and dF/F calculation of calcium imaging data

The baseline estimation was performed using the temporal moving average as follows:

$$MA_{k}=\frac{p_{n-k+1}+p_{n-k+2}+\ldots +p_{n}}{k}\text{.}$$

k is the window length, $p_{n}$ is the fluorescence intensity at time n. Window length was chosen by the recording rate for the data. The resulting dF/F was calculated by subtracting the signal from the baseline and diving by the baseline.

#### Performance metrics

Root-mean-squared error (RMSE), mean absolute error (MAE), peak signal-to-noise ratio (PSNR), structural similarity index measure (SSIM), and Pearson correlation coefficient (PCC) were used as metrics to evaluate the consistency between STABLE translations and references. The RMSE between x and reference y is defined as $\text{RMSE}\left(x,y\right)=\sqrt{E\left[{\left(x-y\right)}^{2}\right]}$, where E denotes the arithmetic mean. The MAE between *x* and reference *y* is defined as $\text{MAE}\left(x,y\right)=\frac{\sum_{i=1}^{n}|y_{i}-x_{i}]}{n}$, where *n* is the number of elements. The PSNR between x and reference y is defined as PSNR(*x, y*) = $10\cdot \log_{10}\frac{{\max\left(x\right)}^{2}}{RMSE{\left(x,y\right)}^{2}}$. The SSIM between x and the reference y is defined as $SSIM\left(x,y\right)=\frac{\left(2\mu_{x}\mu_{y}+C_{1}\right)\left(2\sigma_{xy}+C_{2}\right)}{\left(\mu_{x}^{2}+\mu_{y}^{2}+C_{1}\right)\left(\sigma_{x}^{2}+\sigma_{y}^{2}+C_{2}\right)}$ where $\mu_{x}$ and $\mu_{y}$ are the mean values of x and y, $\sigma_{x}$ and $\sigma_{y}$ are the standard deviations of signal x and y, $\sigma_{xy}$ is the covariance between x and y, and $C_{1}$ and $C_{2}$ are small constants included to stabilize the division when the denominator is close to zero.^53^ The Pearson correlation between x and reference y is defined as $R=\frac{E\left[\left(x-\mu_{x}\right)\left(y-\mu_{y}\right)\right]}{\sigma_{x}\sigma_{y}}$ where $\mu_{x}$ and $\mu_{y}$ are the mean values of x and y, and $\sigma_{x}$ and $\sigma_{y}$ are the standard deviations of x and y.

Centroids of regions of interest (ROIs) were calculated to compare the nearest neighbor distances between nearest translated neurons. The Euclidean distances between the centroids from cytosolic neurons and the centroids from the nearest nuclear-localized neurons were then calculated.

The intersection over union (IoU) scores between the ROIs from cytosolic neurons and its pair of ROIs from nuclear-localized neurons were calculated by creating binary masks and computing the ratio of the intersection area to the union area. The pair was selected by the nearest translated neuron. IoU was defined as the area of overlap divided by area of union.

#### Generation of transgenic zebrafish

The *Tg(HuC:Gal4;UAS:RiboL1-jGCaMP8m)* line was generated using the Tol2 transposon system. The plasmid DNA and *tol2* mRNA were injected in 1-cell stage embryos. We crossed the injected fish with a Casper mutant (*mitfa*(*w2/w2*);*mpv17*(*a9/a9*)) and screened for the neuron-specific expression of green fluorescence in the brain at the F1 generation. The F1 generation is again crossed with a Casper mutant and screened for those expressing green fluorescence and has a Casper phenotype at the F2 generation.

#### *In vivo* calcium imaging of zebrafish brain

For our experiments, we used transgenic larval zebrafish expressing various calcium indicators: GCaMP7a, RiboL1-jGCaMP8m, and H2B-GCaMP6s (Figure S1F). These indicators were controlled by the Gal4-UAS system and the neuron-specific *HuC* promoter, resulting in three specific transgenic lines: *Tg(HuC:Gal4;UAS:GCaMP7a*),^54^^,^^55^^,^^56^ *Tg(HuC:Gal4;UAS:RiboL1-jGCaMP8m*),^33^^,^^57^^,^^58^ and *Tg(HuC:H2B-GCaMP6s*).^37^^,^^59^ The zebrafish used were of the Casper mutant (*mitfa*(*w2/w2*);*mpv17*(*a9/a9*)), and imaging was conducted at 4–5 days post fertilization.

To prepare the larvae for imaging, we first paralyzed them by immersing them in a solution of 0.25 mg/mL pancuronium bromide (Sigma-Aldrich) for 2 min.^60^ Post-paralysis, the larvae were embedded in a 2% low-melting point agarose (TopVision) gel within a Petri dish. Once the agarose solidified, the dish was filled with standard fish water.

For imaging, we used confocal laser scanning microscopes. Two different setups were employed: a Nikon C2+ with a ×16 0.8 NA water dipping objective lens (CFI75 LWD 16X W, Nikon) and a Leica Stellaris 5 with a ×25 0.95 NA water immersion objective lens (HC FLUOTAR L 25×/0.95 W VISIR, Leica). The Nikon system used a 488 nm excitation laser, while the Leica system used a 493 nm excitation laser.

All animal experiments involving zebrafish were conducted in compliance with guidelines and were approved by the Institutional Animal Care and Use Committee (IACUC) of KAIST (approval number KA-2021-125).

#### Two-photon fluorescence and H&E imaging

For the two-photon fluorescence and H&E data imaging, tissue samples from the surgical excision of head and neck tumors were sectioned included various tissue types, such as epithelium, stroma, muscle, and glandular tissues. The tissue sections underwent standard histological paraffin block processing, including formalin fixation, dehydration with ethanol, clearing with xylene, and embedding in paraffin. Once embedded, the paraffin blocks were sectioned at a thickness of 4 μm using a microtome, and the sections were mounted on glass slides. Proximate sections were selected, with one stained with Hematoxylin and Eosin (H&E) and the proximate section imaging using two-photon microscopy (TPM). The H&E-stained sections were obtained using a high-resolution slide scanner. Proximate to the H&E-stained sections, the histological samples were imaged using a commercial two-photon microscope (IVM-CMS, IVIM Technology Inc.) equipped with a 920 nm fs-pulse laser after fluorescence labeling of nuclei, cytoplasm, and extracellular matrix. A 20× objective lens (Plan Apoλ 20X, NA 0.7, Nikon) provided visualization of tissue structures, with an imaging field of view of 0.543 μm/pixel. Afterward, obtained images were separated into two-by-two images. This method enabled direct comparison between the H&E-stained samples and TPM images, ensuring consistency in tissue morphology across different imaging techniques.

#### Comparison with other algorithms

We used publicly available implementations of CycleGAN^25^ (https://github.com/junyanz/pytorch-CycleGAN-and-pix2pix), MUNIT^26^ (https://github.com/NVlabs/MUNIT), SANTA^27^ (https://github.com/Mid-Push/santa), and UTOM^24^ (https://github.com/cabooster/UTOM).

### Quantification and statistical analysis

All statistical analyses were performed using MATLAB 2024a. Detailed statistical information, including the statistical tests used, exact values of n, and what n represents (e.g., number of neurons or cells), can be found in the respective figure captions. In this study, we used two-sample t-tests to assess statistical significance, as described in the figure captions. The exact *p* values and n for each test are also provided in the figure captions.

Measures of central tendency and dispersion, including mean, median, standard deviation (SD), standard error of the mean (SEM), and 95% confidence intervals, are reported in the accompanying tables. Statistical significance was defined as (not significant (n.s.) *p* > 0.05, ∗*p* ≤ 0.05, ∗∗*p* < 0.01, ∗∗∗*p* < 0.001), unless otherwise indicated. The assumptions of normality and equal variance for t-tests were met based on the data distributions.

## Supplemental information

Document S1. Figures S1–S5 and Table S1

Table S2. Excel file containing additional data too large to fit in a PDF, related to Figures 2, 3, 4, and 5

Video S3. Planaria denoising translation, related to STAR Methods

Document S2. Article plus supplemental information
