# Supplementary material for: Preserving spatial and quantitative information in unpaired biomedical image-to-image translation
Source: Cell Rep Methods. 2025 Jun 9;5(6):101074. doi: 10.1016/j.crmeth.2025.101074 (PMC12272260; doi:10.1016/j.crmeth.2025.101074)
Supplement: Document S1. Figures S1–S5 and Table S1 [file mmc1.pdf]

Cell Reports Methods, Volume *5*

Supplemental information

Preserving spatial and quantitative information in unpaired biomedical image-to-image translation

Joshua Yedam You, Minho Eom, Tae-Ik Choi, Eun-Seo Cho, Jieun Choi, Minyoung Lee, Changyeop Shin, Jieun Moon, Eunji Kim, Pilhan Kim, Cheol-Hee Kim, and Young-Gyu Yoon

SUPPLEMENTARY INFORMATION

SUPPLEMENTARY FIGURES

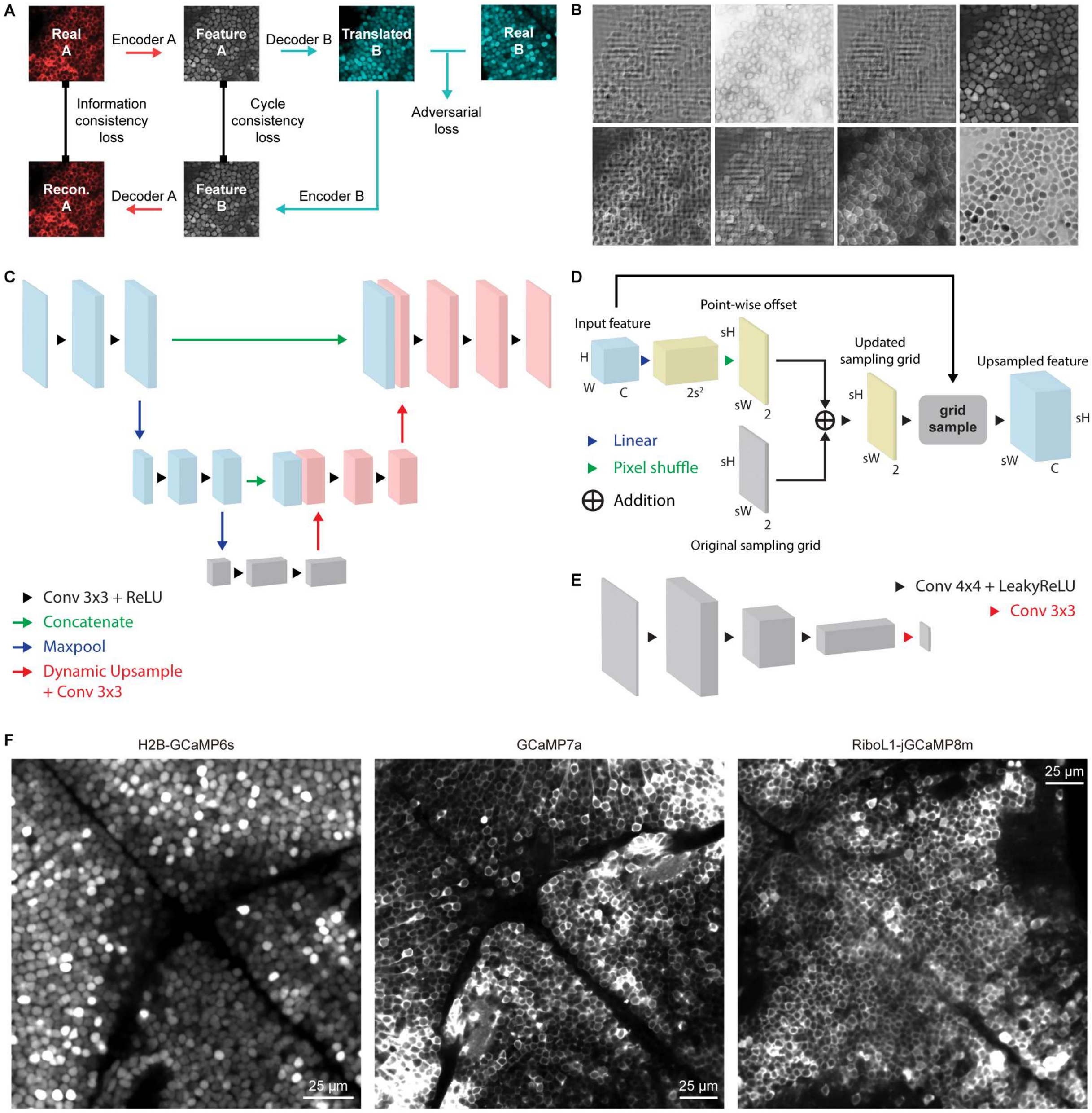

Figure S1. Training pipeline and architectural details of the STABLE network for domain translation. Related to Figure 1, 2, and STAR Methods.

(A) Visualization of the STABLE network's training pipeline for one direction of translation (Domain A to Domain B). In this process, the input image from Domain A (Real A) is passed through Encoder A to extract feature maps (Feature A), which are then decoded by Decoder B to generate the translated image in Domain B (Translated B). A Domain B discriminator evaluates the realism of the translated image compared to a real sample from Domain B, enforcing adversarial loss. The information consistency loss is applied to allow the feature maps from the input image (Real A) and translated images (Translated B) are consistent. Finally, the translated image (Translated B) is passed through Encoder B and Decoder A to reconstruct the original image (Reconstructed A), with cycle

consistency loss allowing that the reconstructed image is consistent with the original. The reverse translation (Domain B to Domain A) follows a similar structure using the corresponding encoders, decoders, and discriminators for the opposite domains.

(B) Visualization of the feature maps extracted from the STABLE network trained on the calcium imaging translation task (Number of channels = 8).

(C) Network architecture of the U-Net based encoder-decoder. The encoder consists of multiple convolutional layers (Conv 3x3 + ReLU) and max-pooling layers for downsampling, extracting hierarchical feature representations. The decoder mirrors this structure, using concatenation (green arrows) to combine encoder features with upsampled features by dynamic upsampling operators (red arrows) for high-resolution image reconstruction.

(D) Network architecture of the dynamic upsampling operator. The input feature map is first passed through a linear convolution layer (blue arrow) followed by a pixel shuffle operation (green arrow) to increase spatial resolution, followed by a point-wise offset that generates an updated sampling grid. This updated grid is then used for resampling the input feature using the grid sample operation.

(E) Network architecture of the discriminator. The discriminator takes an image tensor and applies a series of convolutional layers (Conv 4x4 + LeakyReLU) followed by a final convolutional layer (Conv 3x3), resulting in a single channel output for computing the adversarial loss.

(F) Representative calcium imaging data samples used for training and evaluation of the STABLE network. From left to right: H2B-GCaMP6s (nuclear-localized), GCaMP7a (cytosolic-localized), and RiboL1-jGCaMP8m (cytosolic-localized). These images capture calcium dynamics in distinct cellular compartments—H2B-GCaMP6s shows calcium activity in the nucleus, while GCaMP7a and RiboL1-jGCaMP8m show calcium signals in the cytosol.

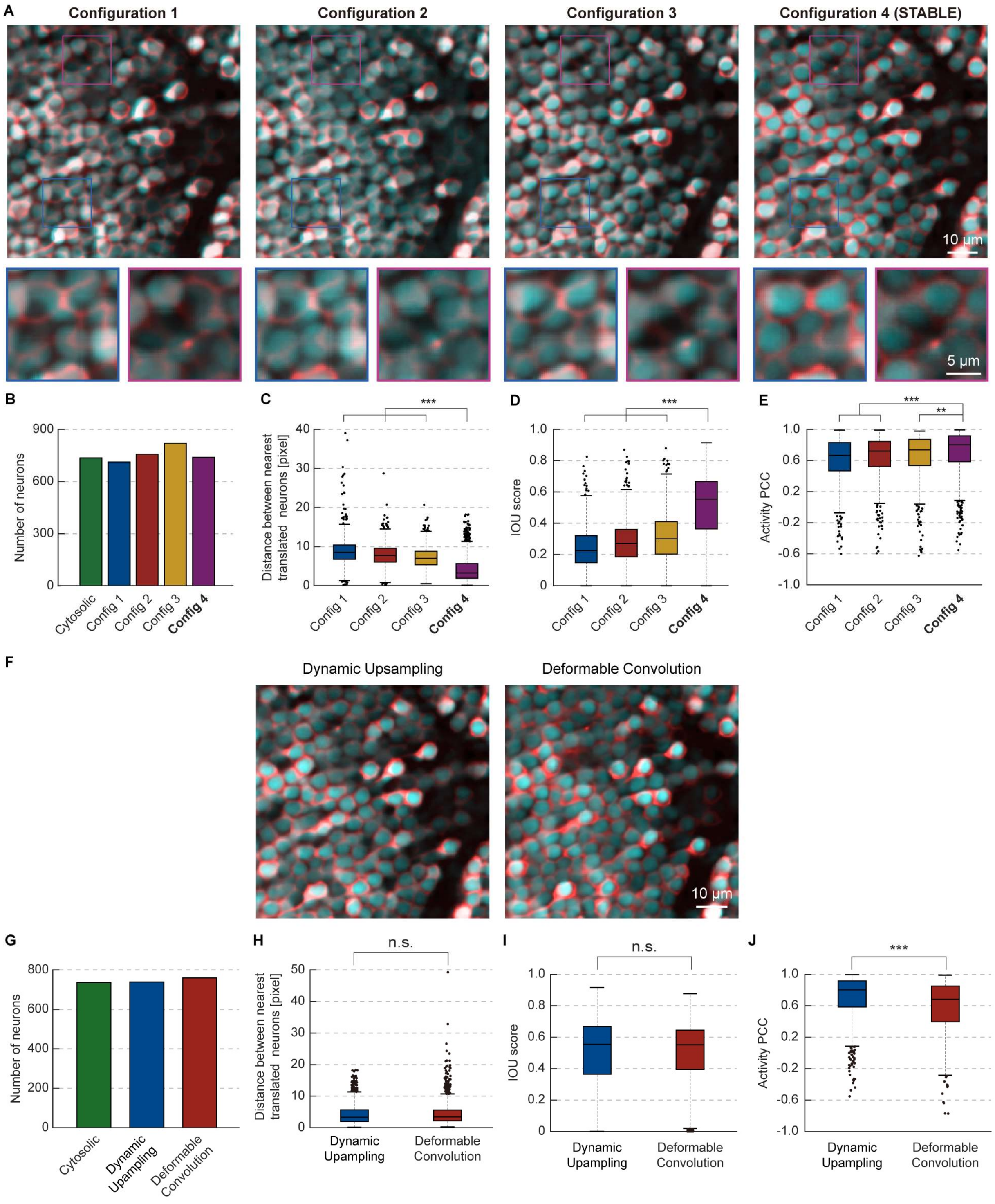

Figure S2. Ablation study on the calcium imaging translation task. Related to Figure 1.

(A) Qualitative results from four different ablation configurations of the STABLE network. Each configuration applies a different combination of adversarial loss, cycle consistency loss, information consistency loss, and dynamic upsampling. The input cytosolic GCaMP image (cyan) is overlaid with the translated nuclear-localized

GCaMP image (red). Magnified views of the boxed regions are presented below for better visualization of differences in translation performance. Configuration 1 (similar to CycleGAN) uses adversarial loss with cycle consistency loss. Configuration 2 incorporates adversarial loss, cycle consistency loss, and dynamic upsampling. Configuration 3 employs adversarial loss, cycle consistency loss, and information consistency loss. Configuration 4 (i.e. STABLE) combines adversarial loss, cycle consistency loss, information consistency loss, and dynamic upsampling.

(B) Comparison of the number of manually segmented neuron regions of interest (ROIs) between the input cytosolic GCaMP and the translated nuclear-localized GCaMP across the four configurations.

(C) Box-and-whisker plot showing the distance between the nearest input and translated neurons for each configuration. The L2 distance between the center of mass of each neuron's segmentation is used for measurement. ($N = 736$ for each test, representing the number of input cytosolic neurons, n.s. $P > 0.05$, *$P \leq 0.05$, **$P < 0.01$, ***$P < 0.001$).

(D) Box-and-whisker plot comparing Intersection over Union (IoU) scores between input and translated ROIs for each configuration. Higher IoU scores indicate better spatial overlap between the input and translated neurons. ($N = 736$ for each test, representing the number of input cytosolic neurons, n.s. $P > 0.05$, *$P \leq 0.05$, **$P < 0.01$, ***$P < 0.001$).

(E) Box-and-whisker plot comparing Pearson correlation coefficients (PCCs) of neuronal activity traces between the nearest input and translated neurons. Higher PCCs represent better preservation of neuronal dynamics during the translation process. A two-sample t-test was performed ($N = 736$ for each test, representing the number of input cytosolic neurons, n.s. $P > 0.05$, *$P \leq 0.05$, **$P < 0.01$, ***$P < 0.001$).

(F) The STABLE implementation with the original grid sampling-based dynamic upsampling operator compared with the deformable convolution-based implementation in the calcium imaging translation task, converting cytosolic GCaMP to nuclear-localized GCaMP. Input cytosolic GCaMP (cyan) is overlaid with the translated nuclear-localized GCaMP (red).

(G–J) Box-and-whisker plots showing the quantitative comparison between the two implementations across multiple evaluation metrics. (G) Number of segmented neuron regions of interest (ROIs), (H) distance between the nearest input and translated neuron ROIs, (I) Intersection over Union (IoU) scores between input and translated ROIs, (J) Pearson correlation coefficients (PCCs) of neuronal activity traces between the nearest input and translated neurons. Two-sample t-tests were performed ($N = 736$ for each test, representing the number of input cytosolic neurons, n.s. $P > 0.05$, *$P \leq 0.05$, **$P < 0.01$, ***$P < 0.001$).).

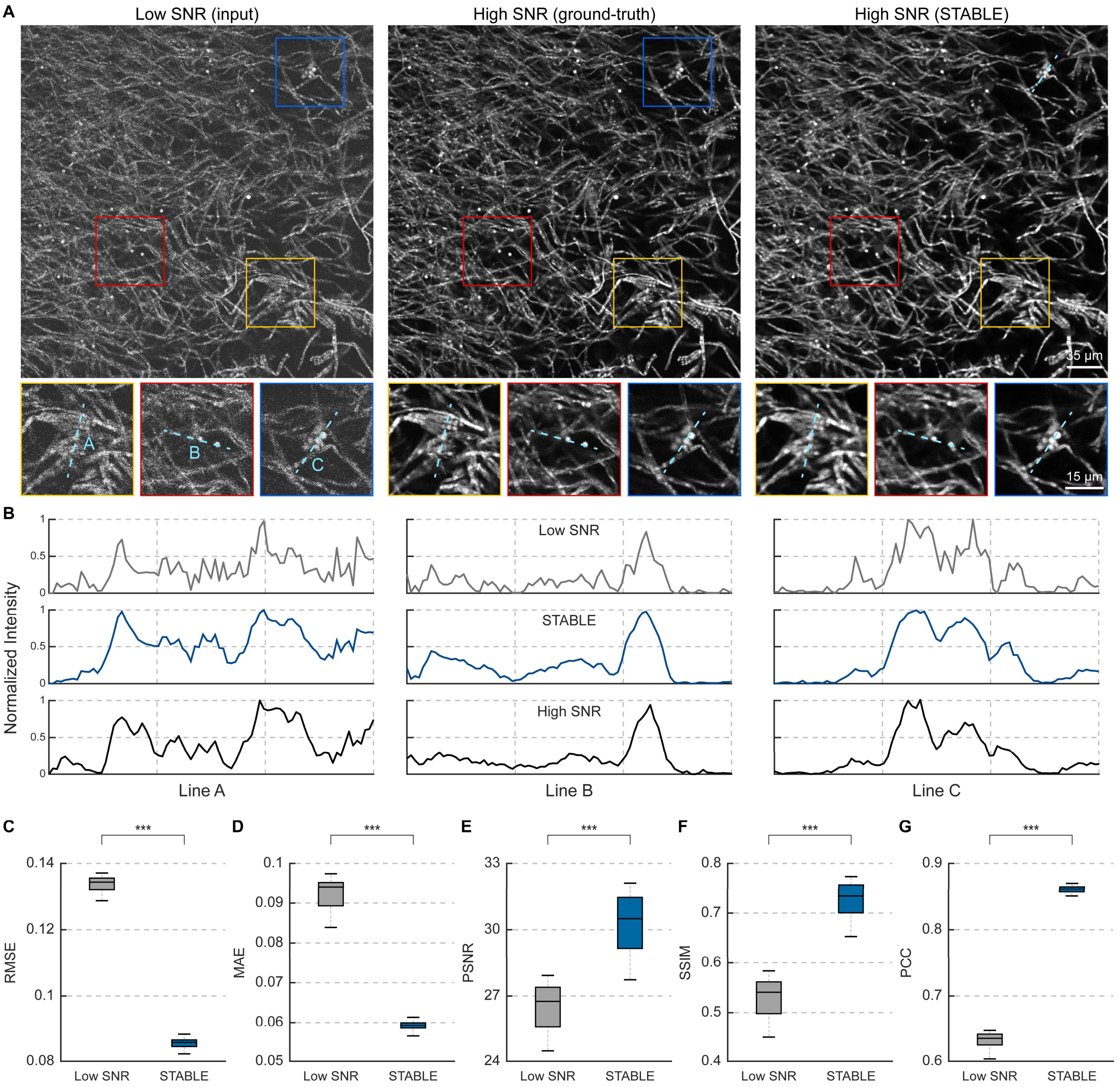

Figure S3. Performance comparison of STABLE in translating low SNR images to high SNR in penicillium imaging data. Related to STAR Methods.

(A) Visualization of the input image with low signal-to-noise ratio (Low SNR input), the high SNR ground-truth (High SNR GT), and the denoised output generated by STABLE. The red, yellow, and blue boxes highlight regions of interest (A, B, and C, respectively), with magnified views shown below, providing a detailed comparison across the three conditions.

(B) Line profiles of normalized intensity across selected regions (Lines A, B, and C). Profiles are shown for the Low SNR input (gray), the output from our method (blue), and the High SNR ground-truth (black).

(C-G) Quantitative evaluation of the denoising performance using various metrics. (C) Root Mean Square Error (RMSE). (D) Mean Absolute Error (MAE). (E) Peak Signal-to-Noise Ratio (PSNR). (F) Structural Similarity Index (SSIM). (G) Pearson Correlation Coefficient (PCC). Two-sample t-tests were performed (N = 100 for each test, representing the number of z-slices used for testing, n.s. P > 0.05, *P ≤ 0.05, **P < 0.01, ***P < 0.001).

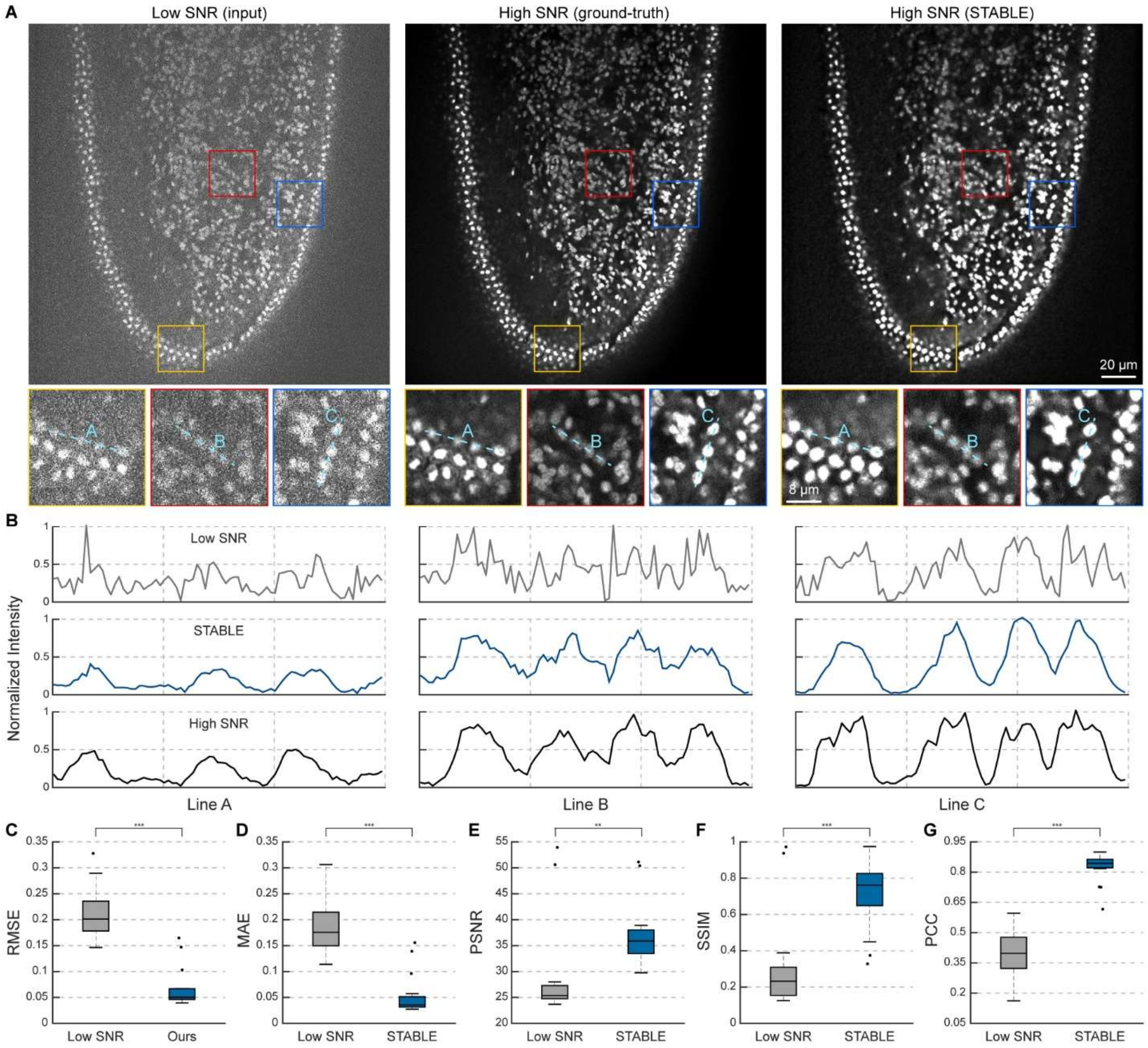

Figure S4. Performance comparison of STABLE in translating low SNR images to high SNR in planaria imaging data. Related to STAR Methods.

(A) Representative images of low signal-to-noise ratio (Low SNR input), high signal-to-noise ratio (High SNR GT), and STABLE's denoised high SNR translation for planarian sample imaging. The red, yellow, and blue boxes highlight regions of interest (A, B, and C, respectively), with magnified views of these regions shown below each image, demonstrating the recovery of fine details by our method in comparison to the Low SNR input.

(B) Normalized intensity profiles along three specific lines (Lines A, B, and C) across the Low SNR input (gray), denoised output from our method (blue), and the High SNR ground-truth (black).

(C-G) Quantitative evaluation of the denoising performance using various metrics. (C) Root Mean Square Error (RMSE). (D) Mean Absolute Error (MAE). (E) Peak Signal-to-Noise Ratio (PSNR). (F) Structural Similarity Index (SSIM). (G) Pearson Correlation Coefficient (PCC). Two-sample t-tests were performed ($N = 20$ for each test, representing the number of volumetric samples used for testing, n.s. $P > 0.05$, *$P \leq 0.05$, **$P < 0.01$, ***$P < 0.001$).

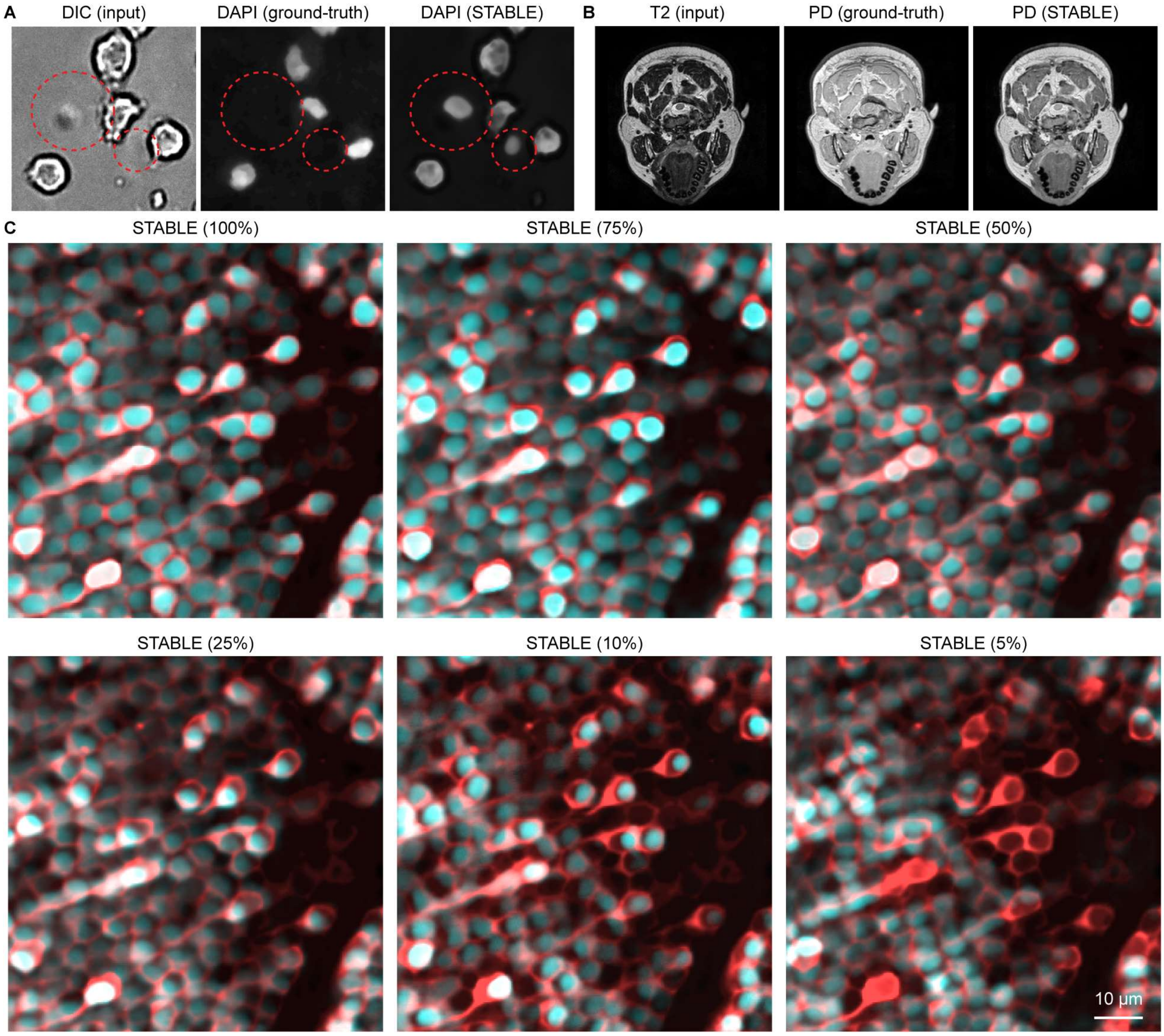

Figure S5. Examples of limitations of STABLE. Related to Figures 1, 4, and 5.

(A) False nuclei generated in the STABLE translated DAPI output correspond to ambiguous structures from the input DIC image.
(B) Slight difference in overall intensity between the STABLE translated PD image and ground-truth.
(C) Effect of training data reduction on STABLE's calcium imaging translation performance. Calcium imaging translation results from STABLE models trained with decreasing proportions of the full dataset: 100%, 75%, 50%, 25%, 10%, and 5%.

TABLES

Table S1. Parameter sensitivity test. Related to Figure 1 and 2.

λ_{adv}	λ_{info}	λ_{cyc}	Number of Z channels	Absolute neuron count difference	Mean distance (pixels)	Median distance (pixels)	Mean IoU	Median IoU	Mean activity PCC	Median activity PCC
0	10	5	8	N/A	N/A	N/A	N/A	N/A	N/A	N/A
2	10	5	8	5	4.239	3.309	0.532	0.500	0.811	0.869
5	10	5	8	4	4.582	3.120	0.567	0.500	0.733	0.802
10	10	5	8	2	7.252	7.008	0.306	0.318	0.629	0.686
100	10	5	8	0	5.545	4.645	0.458	0.430	0.720	0.763
1	0	5	8	38	10.290	9.595	0.173	0.151	0.740	0.785
1	1	5	8	10	8.707	8.410	0.234	0.243	0.732	0.777
1	2	5	8	4	5.044	4.360	0.489	0.452	0.757	0.815
1	5	5	8	4	3.780	2.660	0.611	0.541	0.679	0.776
1	10	5	1	3	9	3.879	2.169	0.577	0.678	0.575
1	10	5	3	9	5.046	3.377	0.498	0.555	0.618	0.705
1	10	5	8	2	3.464	2.601	0.567	0.599	0.800	0.842
1	100	5	8	N/A	N/A	N/A	N/A	N/A	N/A	N/A
1	10	0	8	3	4.014	2.495	0.616	0.536	0.540	0.608
1	10	1	8	0	3.815	2.269	0.624	0.555	0.557	0.651
1	10	2	8	2	4.992	4.003	0.513	0.462	0.759	0.835
1	10	10	8	0	6.683	6.013	0.322	0.313	0.773	0.844
1	10	100	8	10	8.114	8.258	0.282	0.261	0.741	0.814
